# Supplementary material for: Brain responses to food viewing in women during pregnancy and post partum and their relationship with metabolic health: study protocol for the FOODY Brain Study, a prospective observational study
Source: BMJ Open. 2023 Apr 18;13(4):e067013. doi: 10.1136/bmjopen-2022-067013 (PMC10124253; doi:10.1136/bmjopen-2022-067013)
Supplement: Author's manuscript [file bmjopen-2022-067013.draft_revisions.pdf]

BMJ Open

BMJ Open is committed to open peer review. As part of this commitment we make the peer review history of every article we publish publicly available.

When an article is published we post the peer reviewers' comments and the authors' responses online. We also post the versions of the paper that were used during peer review. These are the versions that the peer review comments apply to.

The versions of the paper that follow are the versions that were submitted during the peer review process. They are not the versions of record or the final published versions. They should not be cited or distributed as the published version of this manuscript.

BMJ Open is an open access journal and the full, final, typeset and author-corrected version of record of the manuscript is available on our site with no access controls, subscription charges or pay-per-view fees (http://bmjopen.bmj.com).

If you have any questions on BMJ Open's open peer review process please email info.bmjopen@bmj.com

BMJ Open

Brain responses to food viewing in women during pregnancy and postpartum and their relationship with metabolic health: Study protocol for the FOODY Brain Study

Journal:	*BMJ Open*
Manuscript ID	bmjopen-2022-067013
Article Type:	Protocol
Date Submitted by the Author:	16-Aug-2022

Complete List of Authors:	Lesniara-Stachon, Anna; Lausanne University Hospital, Woman-Mother-Child<br>Quansah, Dan Yedu; CHUV<br>Schenk, Sybille; CHUV<br>Retsa, Chrysa; Laboratory for Investigative Neurophysiology (the LINE), Radiology Department, Lausanne University Hospital and University of Lausanne, Lausanne, Switzerland<br>Halter, Ryan J; Thayer School of Engineering, Dartmouth College, Hanover, NH USA<br>Murray, Micah; Centre Hospitalier Universitaire Vaudois, Departments of Clinical Neurosciences and Department of Radiology<br>Lacroix, Alain; Institute of Higher Education and Research in Healthcare (IUFRS), University of Lausanne, Lausanne, Switzerland.<br>Horsch, Antje; University of Lausanne, Institute of Higher Education and Research in Healthcare; Lausanne University Hospital, Woman-Mother-Child<br>Toepel, Ulrike; Laboratory for Investigative Neurophysiology (the LINE), Radiology Department, Lausanne University Hospital and University of Lausanne, Lausanne, Switzerland<br>Puder, J; Universite de Lausanne, DFME

Keywords:	Diabetes in pregnancy < DIABETES & ENDOCRINOLOGY, Neurobiology < NATURAL SCIENCE DISCIPLINES, NUTRITION & DIETETICS, Eating disorders < PSYCHIATRY

SCHOLARONE™
Manuscripts

TITLE: Brain responses to food viewing in women during pregnancy and postpartum and their relationship with metabolic health: Study protocol for the FOODY Brain Study

Anna Lesniara Stachon [1] (Anna.Lesniara-Stachon@chuv.ch), **Dan Yedu Quansah** [1] (Dan.Quansah@chuv.ch), **Sybille Schenk** [1,5] (Sybille.Schenk@chuv.ch), **Chrysa Retsa** [2,3,4] (chrysa.retsa@gmail.com), **Ryan J. Halter** [6,7] (Alain.Lacroix@chuv.ch), **Micah M. Murray** [2,3,4] (Micah.Murray@chuv.ch), **Alain Lacroix** [8,9] (Alain.Lacroix@chuv.ch), **Antje Horsch** [8,9] (Antje.Horsch@chuv.ch), **Ulrike Toepel** [2] (ulrike.toepel@unil.ch), **Jardena J. Puder** [1] (Jardena.Puder@chuv.ch)

[1] Obstetric Service, Department Woman-Mother-Child, Lausanne University Hospital, Lausanne, Switzerland

[2] Laboratory for Investigative Neurophysiology (the LINE), Radiology Department, Lausanne University Hospital and University of Lausanne, Lausanne, Switzerland

[3] The Sense Innovation and Research Center, Lausanne and Sion, Switzerland

[4] CIBM Center for Biomedical Imaging, Lausanne, Switzerland

[5] Service of Endocrinology, Diabetes and Metabolism, Lausanne University Hospital, Lausanne, Switzerland

[6] Thayer School of Engineering, Dartmouth College, Hanover, NH USA

[7] Department of Surgery, Geisel School of Medicine, Dartmouth College, Hanover, NH, USA

[8] Institute of Higher Education and Research in Healthcare (IUFRS), University of Lausanne, Lausanne, Switzerland

[9] Neonatology service, Department Woman-Mother-Child, Lausanne University Hospital, Lausanne, Switzerland

Correspondence to: Jardena J. Puder, Jardena.Puder@chuv.ch

For peer review only

ABSTRACT

Introduction: Food cravings are common in pregnancy and along with emotional eating and eating in the absence of hunger, they are associated with excessive weight gain and adverse effects on metabolic health including gestational diabetes mellitus (GDM). Women with GDM also show poorer mental health, which further can contribute to dysregulated eating behavior. Food cravings can lead to greater activity in brain centers known to be involved in food 'wanting' and reward valuation as well as emotional eating. They are also related to gestational weight gain. Thus, there is a great need to link implicit brain responses to food with explicit measures of food intake behavior, especially in the perinatal period. The aim of this study is to investigate the spatio-temporal brain dynamics to visual presentations of food in women during pregnancy and in the postpartum, and link these brain responses to the eating behavior and metabolic health outcomes in women with and without GDM.

Methods and analysis: This prospective observational study will include 20 women with GDM and 20 women without GDM. Data will be assessed at 24-32 weeks gestational age and for a subgroup of 14 women in each arm also at 6 months postpartum. The primary outcomes are brain responses to food pictures of varying carbohydrate and fat content during pregnancy and in the postpartum using electroencephalography (EEG). Secondary outcomes including depressive symptoms, current mood, and eating behaviours will be assessed with questionnaires, objective eating behaviours will be

measured using Auracle and stress will be measured with heartrate and heart rate variability (Actiheart). Other secondary outcome measures include body composition and glycaemic control parameters.

Ethics and dissemination: The Human Research Ethics Committee of the Canton de Vaud approved the study protocol (2021-01976). Study results will be presented at public and scientific conferences and in peer-reviewed journals.

For peer review only

For peer review only - http://bmjopen.bmj.com/site/about/guidelines.xhtml

Strengths and limitations of this study:

1. This prospective observational study fills the knowledge gap regarding brain responses to food viewing during and after pregnancy.
2. We will investigate the relationship between spatio-temporal brain dynamics with (pregnancy-related) cravings, disordered eating behaviors and metabolic health.
3. This study includes objective methodologies (EEG, Auracle device, Actiheart) to investigate brain responses to food viewing, eating behaviors and stress responses during and after pregnancy. Linking the outcomes of physiological and behavioral measures is novel.
4. We will compare eating behaviors between healthy women and women with gestational diabetes, both cross-sectionally and prospectively.
5. Limitations include a small sample size (comparable to other EEG studies with a similar design). All analyses (and the correlation of their outcomes) are exploratory, given the lack of data in pregnancy or the postpartum.

For peer review only

INTRODUCTION

Metabolic challenges of pregnancy and the postpartum period

The prevalence of obesity continues to increase worldwide (1). In the United States, 35.7% of women of reproductive age (20-39 years) are obese (2). In high-income western countries, the estimated prevalence of obesity among women of reproductive age exceeds 20% (3).

The widespread and unrestricted access and intake of foods high in sugar and fat contribute to the obesity epidemic. Foods high in sugar and fats, such as chocolate, sweet desserts, and salty snacks are the most frequently craved foods (4).

Although the underlying cause(s) of food cravings are not deeply understood, it has been shown in the general, non-pregnant population that cue-induced cravings in response to food pictures predict disordered eating and excess weight gain. In a meta-analytic review of 45 studies, food cue exposure (pictures) and the experience of craving independently influence eating behavior and weight gain, independent of BMI, age, and gender (5). Maintaining a healthy body weight is particularly important during pregnancy for the health of the mother and her baby (6). However, around 28% of women gain more weight than recommended (7).

During pregnancy, food cravings are frequent: they typically begin at the end of the first trimester, peak in their frequency and intensity in the second and third trimester, and drop after delivery (8). Higher food cravings during pregnancy have been associated with mean energy intake and weight gain (9).

Emotions and stress also influence eating behaviour in pregnancy (10). Emotional cues trigger the craving for high-fat foods, known to be associated with excessive GWG (11). Exposure to higher chronic stress can increase the intake of foods high in fat and sugar (12), especially among overweight individuals (13,14).

The relationship between GWG and adverse obstetric and metabolic health outcomes in the postpartum period highlights the need for novel approaches to understand eating behaviour and excess weight gain during pregnancy and the postpartum, especially in metabolic high-risk women with GDM (6).

Relationship between brain responses and metabolic health

The few existing clinical data show that reward-related brain regions may be involved in excess weight gain (15), because craved foods stimulate the brain reward and motivation pathways and through unconscious processes of learning/conditioning mechanisms, promote greater 'wanting' and seeking for craved foods (16). Increased neural reactivity to food cues in reward-related brain regions promotes food cravings (17,18). Food cues and sensory stimulation also increase food cravings and food intake whether in a hungry or satiated state (19).

Higher intake of foods rich in fat and sugar can reduce reward thresholds in the limbic striatal pathways and the amygdala (17,18,20). This then promotes further food craving, leading to increased neural reactivity to food cues in reward-related brain regions, promoting a higher intake of palatable foods (17,18).

Food cravings and overeating in response to stress and negative mood are particularly relevant in emotional eaters (21, 44, 45). In a functional magnetic resonance imaging (fMRI) study, emotional eaters responded with stronger responses to high-calorie food under negative mood in reward-associated areas (anterior cingulate cortex) in comparison to non-emotional eaters (47).

Response to food viewing in regions linked to cognitive evaluation processes and rewarding stimuli differ between obese and healthy weight subjects. Overweight participants also demonstrated greater activation in reward-related brain regions, like the ventral striatum and anterior cingulate (23), and lower activation in brain regions associated with cognitive control (24) compared to normal weight and successful weight-losing adolescents. Adolescents who successfully lost and maintained weight had greater activation of the dorsolateral prefrontal cortex, responsible for cognitive control, in response to high-energy food images compared to overweight or normal-weight adolescents (23).

Insulin resistance and type 2 diabetes mellitus (DM2) are associated with changes in brain responses to food stimuli. FMRI studies revealed that responses to viewing food pictures among patients with DM2 are stronger in the basal ganglia and cortical regions, such as the insula and the orbitofrontal cortex, related to reward-related circuits, in comparison to non-diabetic controls (22). These results demonstrate the link between brain responses to food viewing and metabolic health and the role of reward-and cognitive control-related brain regions with food intake regulation.

EEG and visual evoked potentials (VEPs; patterns of electrical activity in the brain in response to a visual stimulus) to food viewing offer the advantage of disentangling the temporal as well as spatial dynamics of perception, while fMRI only unravels spatial aspects, i.e., brain regions. Some VEP studies have shown differential responses to pictures of high and low-energy foods (25). Reward- and (inhibitory) control-related processes are partially recruiting the same neural sources, yet with varying temporal dynamics (48).

Existing studies on food cravings during and after pregnancy only assessed different kinds of food cravings and their frequency with questionnaires and did not involve brain responses to food cues. Results from these studies have been inconsistent. We are not aware of any human studies investigating objective correlates or mechanistic pathways of cravings in pregnancy. In order to better understand food cravings and metabolic health outcomes in pregnancy and postpartum, investigating the neural activities of the brain pathways in response to cravings induced by food viewing during and after pregnancy might lead to novel clinical treatment options.

Aims of the present study

The overall aim of this observational study is to investigate brain responses to viewing pictures of foods of different fat and carbohydrate contents in women during pregnancy and in the postpartum

For peer review only - http://bmjopen.bmj.com/site/about/guidelines.xhtml

and their relationship with eating behaviour and metabolic health. We will investigate this in healthy women and in a metabolic high-risk group, i.e., women with GDM.

The main objectives are:

Objective 1: To investigate differences in brain responses to food pictures of varying fat and carbohydrate content (HIGH-FAT/LOW-CARB; HIGH-FAT/HIGH-CARB; LOW-FAT/LOW-CARB; LOW-FAT/HIGH-CARB) during and after pregnancy, in women with and without GDM.

Objective 2: To investigate the cross-sectional and longitudinal relationships between brain responses to food viewing with reported food cravings, eating behaviour, and metabolic health outcomes during pregnancy and in the postpartum in women with and without GDM.

Objective 3: To investigate differences in brain responses to food pictures in relationship to different emotional states and stress levels.

METHODS

Study design

This is a prospective observational study of women with and without GDM. There will be two visits (at 24-32 weeks gestational age and 6 months postpartum). Both visits will take place at Lausanne University Hospital.

Study population, inclusion and exclusion criteria, and recruitment

We will recruit 20 women with GDM and 20 healthy controls, in their 24-32 weeks of pregnancy, during their antenatal appointments visits at a Swiss University Hospital. GDM women (clinical group) will be recruited at the GDM clinic whereas healthy pregnant women without GDM (control group) will be recruited at the hospital's ambulatory clinic.

For women with GDM, inclusion criteria are: gestational age 24- 32 weeks, age ≥ 18 years, GDM diagnosed at 24-32 gestational weeks according to IADPSG and ADA criteria (26,27). For healthy controls, inclusion criteria include women with normal weight (pre-pregnancy BMI < 25 kg/m2), with no prior or current GDM, no diets and weight changes above 5 kg in the last 5 years (except for pregnancy), no excessive pregnancy weight changes (less than twice of the Institute of Medicine recommendations (28)).

Exclusion criteria include: pre-existing diabetes, eating disorders (Anorexia Nervosa, Bulimia Nervosa, and Binge-Eating Disorder), vegetarian/vegan diet, uncontrollable nausea and vomiting, current insulin treatment, participation in an intervention study, current medication to treat psychiatric disorders, active suicidal thoughts.

Primary outcome

The primary outcomes are EEG viewing responses, specifically to food images depending on fat and carbohydrate content (HIGH-FAT/LOW-CARB; HIGH-FAT/HIGH-CARB; LOW-FAT/LOW-CARB; LOW-FAT/HIGH-CARB). These responses will be examined in terms of amplitude and latency of the brain responses (VEPs) as well as in terms of response topography. An estimation of the underlying intracranial sources of the VEPs will also be performed. The full analysis pipeline is referred to as "Electrical Neuroimaging" as has been developed and validated by members of our team and their collaborators (for example, Michel and Murray, 2012; Murray et al., 2008; Brunet et al., 2011). VEPs will be compared during (visit 1) and after (visit 2) pregnancy, as well as between women with and without GDM).

For peer review only - http://bmjopen.bmj.com/site/about/guidelines.xhtml

Secondary outcomes

Secondary outcomes are weight, HbA1c, glucose measures at visits 1 and 2 (the latter only in women with GDM) and body composition (fat/lean mass). We will investigate eating rate, objectively using a novel eating recognition device (Auracle) and subjectively (reported perceived eating rate). Using validated self-report questionnaires we will assess eating behaviours, including cravings (Food Craving Questionnaire-Trait-reduced), intuitive eating (IE) (The French Intuitive Eating Scale-2), external, emotional eating, and cognitive restriction (The Three-Factor Eating Questionnaire). Depression scores will be assessed by Edinburgh Postnatal Depression Scale and current mood by PANAS questionnaire Positive and Negative Affective Schedule. Other measures will include self-reported stress level assessed with a questionnaire (The Perceived Stress Scale) and objectively measures of stress using Actiheart.

Data collection and visits

The first visit will take place during pregnancy, at 24-32 weeks gestational age for all women (visit 1). A subpopulation of 14 women in each group will be invited to participate also in the second visit at 6 months postpartum (visit 2).

After signing the informed consent, and before visit 1 and visit 2, participants will complete validated questionnaires online (see below). They will be contacted by phone to complete missing data regarding their medical history as well as their lifestyle behaviours.

Before each visit, participants will be asked to eat a balanced meal around 12.00 (based on the Swiss Society for Nutrition recommendations), will come to the hospital at 13h30 and testing will be completed at 16h (Table 1).

For peer review only - http://bmjopen.bmj.com/site/about/guidelines.xhtml

Flowchart of the study procedure:

			Visit 1	Visit 2
	Study schedule			
Time point			3rd trimester of pregnancy	6 month postpartum
Duration			Tele-visit: 10-30 min for medical history and 20-40 min for online questionnaires<br><br>Visit: max 2h 30 min including breaks*	Tele-visit: 10-30 min for medical history and 20-40 min for online questionnaires<br><br>Visit: max 2h 30 min including breaks*
Procedures and time				
**Tele-visit before or after each of both of the physical visits (at home)**				
Medical history if lacking in the chart and questions regarding lifestyle, by phone		10-30 min		
Questionnaires online: Food Craving Questionnaire-Trait-reduced, Edinburgh Postnatal Depression Scale, Edinburgh Handedness Inventory, TFEQ - R18, Intuitive Eating Scale – 2		20-40 min	X	X
**During the visit (in the EEG lab up to the break)**				
Set-up EEG, questionnaires:<br><br>Perceived Stress Scale (PSS-10)<br>Food Craving Questionnaire-State<br>PANAS questionnaire: Positive and Negative Affective Schedule,<br>current hunger level: Likert scale) + heart rate (Actiheart)	13h30-14h15	30 - 45 min	X	X

For peer review only - http://bmjopen.bmj.com/site/about/guidelines.xhtml

Electroencephalography	14h15-15h10	55 min	X	X
Break	15h10-15h20	10 min	X	X
Bioimpedance & weight	15h20-15h30	10 min	X	X
Positive and Negative Affective Schedule current hunger level: Likert scale<br><br>Break and Auracle* + questionnaire: Eating of the provided snacks & 3 questions regarding eating duration	15h30-15h45	15 min	X	X
Height, blood pressure	15h45-15h50	5 min	X	X
Capillary measure: HbA1c Double check lunch composition	15h50-15h55	5 min	X	X
Set-up glucose sensor: Freestyle Libre 1 (only for GDM) x 14 days	15h55-16h00	5 min	X	X

Table 1: Study schedule.

**During the Auracle session, patients will eat different snacks that we provide.*

Patient and Public Involvement

The development of the research question and outcome measure was also based on remarks from patients in clinical settings, we did not involve patients significantly in the study design. Some results will be provided to participants at the end of their study as feedback by sending them to their healthcare providers. Interested patients will also receive an overall return of the results.

MEASURES

Behavioural task/Electroencephalography

We will employ a behavioural task involving food pictures of variable fat and carbohydrate content and will record high-density VEPs (brain responses to visual stimuli via electroencephalography (EEG)) (figure 1). Using the Salzburg food picture database (29), we are going to show 4 blocks of 240 randomized images (200 pictures of food and 40 non-food objects together; 50 pictures of food and 10 non-food objects per block) to the participants. The food images are divided into 4 categories: HIGH-FAT/LOW-CARB; HIGH-FAT/HIGH-CARB; LOW-FAT/LOW-CARB; LOW-FAT/HIGH-CARB.

HIGH-FAT was defined by a fat content of > 5 g/100g (21.6 +/- 13.77 g/100g) and LOW-FAT of < 5 g/100g (mean 1.1 +/- 1.29 g/100g). Similarly, HIGH-CARB was defined as >15g/100g carb (48.57 +/-

For peer review only - http://bmjopen.bmj.com/site/about/guidelines.xhtml

21.13 g/100g) and LOW-CARB as < 15 g/100g (4.59 +/- 4.59 g/100g). We aimed for the contents to be as far as possible away from the cut-off to provide the best distinction.

Images will be identical in size, controlled for low-level visual features (like luminance), and will be presented on the computer monitor during EEG recording sessions. Immediately after the presentation of each image, women have to decide if the presented picture is a food or non-food object by pressing a button 1 or 2 on a response box. Accuracy and reaction time of behavioural responses will be collected. At the beginning of the experimental session, the participant will be presented with the instructions and will have to perform some trials (7 pictures of food and 3 of non-food) to familiarize herself with the task. Continuous 128-channel EEG will be recorded on the head surface. VEPs will be calculated separately for each image category (low-fat/low-carb; low-fat/high-carb; high-fat/ low-carb; high-fat/high-carb). The preparation for the EEG recording will take approximately 30-45 min, whilst EEG sessions will last maximum 55 minutes. When the EEG experiment is completed, the patient will move to the preparation room for the validation task. Twenty pictures of food (5 pictures from each food category) will be presented and the patient will be asked to rate how much she wants this food.

Heart rate and heart rate variability

During EEG and the Auracle testing, we will monitor the participant's heart rate and heart rate variability to assess the level of arousal of the autonomic system. We will perform baseline and recovery measurements (2 min each) before and after EEG and the Auracle testing. For this purpose, we will use Actiheart, a compact chest-worn monitoring tool that apart from heart rate, measures Inter-Beat-Interval (IBI). Specific time points before and after the test will be identified by a stopwatch. To analyze the data, we will use the commercial Actiheart Software.

Body composition

Body composition will be measured by bioimpedance (BIA 101, Akern, Italy) at both visits. Using four electrodes (two on the right hand and two on the right foot, at a distance of 3–4 cm from each other) and low-voltage electric flows, we will measure the resistance (Rz) and reactance (Xc) of tissue to assess the fat and lean mass.

Eating behavior ("Auracle" and choice of snacks)

The Auracle (see Figure 2) will be used as an objective measure of eating behaviour (see Figure 3 and below). Auracle is an eating-recognition system that measures vibrations generated from movement, chewing and teeth grinding. When worn, the contact microphone is positioned on the mastoid and when a person eats, vibrations from movement, chewing and grinding of teeth sensed by the device are transmitted and stored on an embedded memory card. This device automatically identifies the eating rate and rapidity as well as the duration of food intake and other related dynamics. Auracle locally captures, processes, and classifies sensor data collected during eating periods. All of the sensors in Auracle (ADXL362 accelerometer and CM-01B microphone) are commercial, off-the-shelf devices. The device includes an MSP430 microcontroller (Texas Instruments, Dallas TX), a push button and buzzer for user interaction. It is comfortable to wear because it is embedded in an elastic headband or a flexible plastic frame and possesses no risk to the mother or fetus.

Eight different crunchy snacks of different carbohydrate and fat content (2 of each corresponding to one of the four categories described in the EEG part) will be offered (Figure 3). As eating behaviour will be measured with the "Auracle" device, crunchy-textured snacks provide a stronger, more easily detected vibrational signal. Before the snack session, the patient will be asked the second time about

For peer review only - http://bmjopen.bmj.com/site/about/guidelines.xhtml

their current level of hunger (1- not at all to 5- very hungry) and fill out the "PANAS" questionnaire. The weight of the different snacks will be measured before and after the patient has finished eating to determine the number of kcal and the quantity of fat, protein and carbohydrates eaten. Furthermore, the time interval between the first and last bite will be also measured using the "Auracle" device and a stopwatch. We will also ask the woman questions regarding her subjective perceived eating rate (perceived eating duration, perceived eating speed and current eating speed compared to usual eating speed).

Collection of blood samples

Capillary blood will be collected to measure HbA1c level. HbA1c will be analyzed by Afinion®.

Glucose sensors

Women with GDM will use Freestyle Libre 1 glucose sensors (Abbott®) for flash glucose measurement for 14 days. Sensors will be applied on the back of the arm, with a sterile fiber being inserted under the skin into the interstitial fluid. Patients use a dedicated application LibreLink and will have to scan the glucose sensor at least every 8h to transfer data. Data will be linked in a coded form to the online platform Libreview (https://www.libreview.com/). During pregnancy, results will be discussed with their health care provider during the visits. For the postpartum visit, patients will receive feedback by phone.

Questionnaires related to eating behavior and mental health

Women with and without GDM will fill out the same questionnaires during both visits (visit 1 & 2). Most women will fill out the questionnaires in French and a few in English. The following questionnaires will be filled out online before the study visits:

The French Intuitive Eating Scale-2 (IES-2) (30): Validated self-report questionnaire that assesses individuals' tendency to follow their physical, hunger and satiety cues in determining when, what and how much to eat. The original version (English) consists of 4 subscales, Eating for physical rather than emotional reasons (EPR, 8 items), Reliance on hunger and satiety cues (RHSC, 6 items), Unconditional permission to eat (UPE, 6 items) and the Body-Food Choice Congruence (BFC-C, 3 items) subscales. The validated French version however consists of 18-items divided into three subscales, the EPR (8-items), RHSC (6-items) and the UPE (4-items) subscales (31), and has also demonstrated good psychometric properties. In this study, we will assess intuitive eating with only the EPR and the RHSC subscales (14-items) of the French-adapted version of IES-2 and the English version. Scores of IES-2 range between 1 and 5. A higher score of the EPR subscale reflects eating as an answer to hunger and a lower score means eating to cope with emotional distress, whereas a higher score on the RHSC subscale signifies trust in internal cues, and a lower score reflects less ability to regulate food intake.

The Three-Factor Eating Questionnaire-Revised 18 (TFEQ-R18): 18-item self-report questionnaire that measures three aspects of eating behaviours including cognitive restraint (6 items), uncontrolled eating (9 items), and emotional eating (3 items). Items are scored on a Likert scale ranging from 1 'Almost always' to 4 'Never'. A short, revised version of the Stunkard and Messick TFEQ was developed by Karlsson et al. (32). It has been also tested in the general population in France (33).

Food Craving Questionnaire-Trait-reduced (FCQ-T-r) (34): 15 item self-report questionnaire for the assessment of the frequency and intensity of food craving. It consists of 5 subscales: lack of control over eating (5 items), thoughts or preoccupation with food (5 items), intentions and plans to consume food (2 items), emotions before or during food craving (2 items), and cues that may trigger food craving (1 item). Items are scored on a Likert scale from 1 'Never/not applicable' to 6 'Always'. Higher scores

For peer review only - http://bmjopen.bmj.com/site/about/guidelines.xhtml

reflect food-cue elicited craving. The French version was validated and has shown good psychometric proprieties (35).

Edinburgh Postnatal Depression Scale (EPDS) (36): 10-item self-report questionnaire to examine postnatal depression symptoms over the previous week, which are scored on a 4-point Likert scale. Scores range from 0 to 30. Higher scores indicate a higher risk of postnatal depression. The French version of the EPDS demonstrated good psychometric properties (37).

Edinburgh Handedness Inventory short form (38): This short (4 items) and simple questionnaire measures the preference to use one hand more than the other. The French version has been previously used in research (39).

During the study visits, participants will complete the following questionnaires:

The Positive and Negative Affective Schedule questionnaire (PANAS) (40): Self-report questionnaire that assesses mood or emotion during the last month. It consists of 20 items, 10 items measuring positive affect (e.g., enthusiastic, proud) and 10 items measuring negative affect (e.g., scared, ashamed), which are scored on a Likert scale from 1 'Not at All' to 5 'Extremely'. Individual scores on the PANAS range from 10 to 50, with higher scores representing higher levels of positive affect. The validated French version has shown good psychometrics (41).

The Perceived Stress Scale (PSS-10): 10 item questionnaire widely used to assess stress levels during the last month. Responses are rated on a Likert scale from 0 'Never' to 5 'Very often'. The results range from 0 to 40, with higher scores indicating higher perceived stress. The French version of PSS-10 has shown good psychometric properties (42).

The Food Craving Questionnaire-State (FCQ–S) (43): 15 item questionnaire measures the intensity of momentary food cravings. Items are scored on a 5-point scale ranging from 'strongly disagree' to 'strongly agree'. Higher scores indicate more intense current food cravings. The French version was translated and used in previous research by the research team of our collaborator Ulrike Toepel (39).

The current hunger level scale: This is one item developed for this study assessing the current perceived hunger level using a Likert scale ranging from 1 'just a little/not at all' to 5 'very much'.

The food wanting questionnaire: 20-item questionnaire was developed for this study and will be completed after the EEG (see above). Women will be shown 20 pictures of food and asked 'How much do you want this food?'. They will then have to indicate their response on a Likert scale from 0 'not at all' to 10 'very much'.

DATA MANAGEMENT AND STATISTICAL ANALYSES

Statistical analysis plan

The sample size is based on previous EEG studies of similar design (44,45), but all analyses are exploratory, as to our knowledge so far no such study has been conducted during pregnancy or the postpartum period. Statistical analyses will be performed with STATA version 15.1 (2017) and MATLAB version 9.5 (R2018b). Processing of the EEG data will be performed using Cartool software and statistical software (46). Demographic and other descriptive variables will be represented as means and standard deviations or percentages where appropriate. ANOVA, t-tests or Chi-square tests will be used to determine group differences between responses to food and non-food pictures, different food categories, brain responses between pregnancy and postpartum states, and between women with and without GDM. Regression analyses will be used to model the relationships between predictor variables and outcomes (such as between brain responses to food viewing with intuitive

For peer review only - http://bmjopen.bmj.com/site/about/guidelines.xhtml

eating and metabolic health). In the second step, we will investigate the impact of covariates, such as maternal age, gestational age, and pre-pregnancy BMI on outcomes and adjust for them if necessary.

Twenty women in each group (n=40) will be investigated during pregnancy and a subgroup of 14 in each group are invited to participate during both visits, i.e during pregnancy and at 6 months postpartum.

As pregnancy and the postpartum period is a time where drop-outs are frequent, we assume an attrition rate of 33% and will include 28 women total for both visits to reach the complete recruitment target for the postpartum visit (10 women with GDM and 10 healthy women for both visits taking the estimated attrition into account).

Ethics and dissemination

The local ethical committee approved the study protocol (study number 2021-01976). The study poses little to no risk to participants. Signed informed consent will be obtained from all participating women. The results of the study will be disseminated through national and international conferences, in peer-reviewed journals and presented at public conferences and other scientific meetings.

Significance and outlook

The prevalence of obesity continues to increase worldwide. This study seeks to understand brain reactions to the viewing of food images and their changes during pregnancy and in the postpartum period. Food cravings and disordered eating behaviors include implicit processes in the brain. Studying these processes could help unravel mechanistic pathways associated with weight gain. Our study may provide novel evidence of scientific and clinical importance to understand eating behaviour, potentially opening perspectives for future interventions that alter reactions to energy-dense food in general or at specific food nutrients and/or brain regions to manage excessive GWG, weight retention, and to reduce adverse metabolic outcomes in the postpartum period. It is a potential opportunity to reduce craving, incentive sensitization and thus the overstimulation of reward circuits by food cues in pregnant and postpartum states where a dynamic change in craving and weight gain is observed. The study aims to provide data for a better understanding and long-lasting interventions that can influence the health of the mother and the child.

Author affiliations

[1] Obstetric Service, Department Woman-Mother-Child, Lausanne University Hospital, Lausanne, Switzerland

[2] Laboratory for Investigative Neurophysiology (the LINE), Radiology Department, Lausanne University Hospital and University of Lausanne, Lausanne, Switzerland

[3] The Sense Innovation and Research Center, Lausanne and Sion, Switzerland

[4] CIBM Center for Biomedical Imaging, Lausanne, Switzerland

[5] Service of Endocrinology, Diabetes and Metabolism, Lausanne University Hospital, Lausanne, Switzerland

[6] Thayer School of Engineering, Dartmouth College, Hanover, NH USA

[7] Department of Surgery, Geisel School of Medicine, Dartmouth College, Hanover, NH, USA

[8] Institute of Higher Education and Research in Healthcare (IUFRS), University of Lausanne, Lausanne, Switzerland.

For peer review only

[9] Neonatology service, Department Woman-Mother-Child, Lausanne University Hospital, Lausanne, Switzerland.

Author contributions JP designed the study with input from all co-authors, particularly DQ. CHR, UT and MMM contributed to the creation and design of the EEG section, along with SS, DQ, ALS and JP. SS was responsible for the nutritional part regarding the lunch and snacks. AH contributed to the psychology part and AL, along with DQ, to the statistical and data analysis part. RJH was responsible for the design of "Auracle", adapting it together with DQ. ALS wrote the first draft and all authors critically revised the final version of the manuscript.

Funding This work was supported by a donation from the Dreyfus foundation and an unrestricted educational grant from NovoNordisk N/A. Ryan Halter's research program is supported by the US National Science Foundation under award numbers CNS-1565269, CNS-1835983, CNS-1565268, and CNS-1835974.

Competing interest None declared.

Patient consent for publication Not required.

Ethics approval Commission d'Ethique du Canton de Vaud, Switzerland, study number: 2021-01976

Open access This is an open access article distributed in accordance with the Creative Commons Attribution Non Commercial (CC BY-NC 4.0) license, which permits others to distribute, remix, adapt, build upon this work non-commercially, and license their derivative works on different terms, provided the original work is properly cited, appropriate credit is given, any changes made indicated, and the use is non-commercial. See: http://creativecommons.org/licenses/by-nc/4.0/.

Figure legends

Figure 1: Electroencephalography, Figure 2: The Auracle, Figure 3: Snacks

For peer review only

REFERENCES

1) NCD Risk Factor Collaboration (NCD-RisC). Worldwide trends in body-mass index, underweight, overweight, and obesity from 1975 to 2016: a pooled analysis of 2416 population-based measurement studies in 128·9 million children, adolescents, and adults. Lancet 2017;390(10113):2627–42.

2) Hales CM, Fryar CD, Carroll MD, Freedman DS, Ogden CL. Trends in Obesity and Severe Obesity Prevalence in US Youth and Adults by Sex and Age, 2007-2008 to 2015-2016. JAMA. 2018;319(16):1723–1725. doi:10.1001/jama.2018.3060

3) NCD Risk Factor Collaboration, Heterogeneous contributions of change in population distribution of body mass index to change in obesity and underweight, eLife, 2021

4) Gilhooly CH et al. Food cravings and energy regulation: the characteristics of craved foods and their relationship with eating behaviors and weight change during 6 months of dietary energy restriction. Int J Obes (Lond). 2007.

5) Boswell RG, Kober H. Food cue reactivity and craving predict eating and weight gain: a meta-analytic review. Obes Rev. 2016 Feb;17(2):159-77. doi: 10.1111/obr.12354. Epub 2015 Dec 8. PMID: 26644270; PMCID: PMC6042864.

6) Kominiarek MA, Peaceman AM. Gestational weight gain. Am J Obstet Gynecol. 2017;217(6):642-651.

7) Martínez-Hortelano, J.A., Cavero-Redondo, I., Álvarez-Bueno, C. et al. Monitoring gestational weight gain and prepregnancy BMI using the 2009 IOM guidelines in the global population: a systematic review and meta-analysis. BMC Pregnancy Childbirth 20, 2020

8) Belzer LM, Smulian JC, Lu SE, Tepper BJ. Food cravings and intake of sweet foods in healthy pregnancy and mild gestational diabetes mellitus. A prospective study. Appetite. 2010;55(3):609–15.

9) Hill AJ, McCance DR. Anthropometric and nutritional associations of food cravings in pregnancy. Pregnancy Hypertens. 2014

10) M.W. Chang, S. Nitzke, E. Guilford, C.H. Adair, D.L. Hazard. Motivators and barriers to healthful eating and physical activity among low-income overweight and obese mothers Journal of the American Dietetic Association, 2008

11) Blau, L. E., Orloff, N. C., Flammer, A., Slatch, C., & Hormes, J. M. (2018). Food craving frequency mediates the relationship between emotional eating and excess weight gain in pregnancy. Eating behaviors, 31, 120-124. https://doi.org/10.1016/j.eatbeh.2018.09.004

12) Barrington WE, Beresford SA, McGregor BA, White E: Perceived stress and eating behaviors by sex, obesity status, and stress vulnerability: findings from the vitamins and lifestyle (VITAL) study. J Acad Nutr Diet 2014;114:1791–1799.

13) Lemmens SG, Rutters F, Born JM, Westerterp-Plantenga MS (2011): Stress augments food ‘wanting’ and energy intake in visceral overweight subjects in the absence of hunger. Physiol Behav

14) Warne JP. Shaping the stress response: Interplay of palatable food choices, glucocorticoids, insulin and abdominal obesity. Molecular and Cellular Endocrinology. 2009

For peer review only - http://bmjopen.bmj.com/site/about/guidelines.xhtml

15) Frankort A, Roefs A, Siep N, Roebroeck A, Havermans R, Jansen A. The craving stops before you feel it: Neural correlates of chocolate craving during cue exposure with response prevention. Cereb Cortex. 2014

16) Alsiö J, Olszewski PK, Levine AS, Schiöth HB. Feed-forward mechanisms: Addiction-like behavioral and molecular adaptations in overeating. Frontiers in Neuroendocrinology. 2012.

17) Sinha R. Role of addiction and stress neurobiology on food intake and obesity. Biol Psychol. 2018

18) Cottone P, Sabino V, Roberto M, Bajo M, Pockros L, Frihauf JB, et al. CRF system recruitment mediates dark side of compulsive eating. Proc Natl Acad Sci U S A. 2009;106(47):20016–20.

19) Mccrickerd K, Forde CG. Sensory influences on food intake control: Moving beyond palatability. Obes Rev. 2016

20) Ghitza UE, Gray SM, Epstein DH, Rice KC, Shaham Y. The anxiogenic drug yohimbine reinstates palatable food seeking in a rat relapse model: a role of CRF1 receptors. Neuropsychopharmacology. 2006;31(10):2188-2196. doi:10.1038/sj.npp.1300964

21) Yau Y. H., Potenza M. N. (2013). Stress and eating behaviors. Miner. Endocrinol. 38:255.

22) M. Chechlacz, Diabetes dietary management alters responses to food pictures in brain regions associated with motivation and emotion: a functional magnetic resonance imaging study, Diabetologia, 2009

23) Jensen CD, Kirwan CB. Functional brain response to food images in successful adolescent weight losers compared with normal-weight and overweight controls. Obesity (Silver Spring). 2015

24) Samantha J. Brooks, Increased Prefrontal and Parahippocampal Activation with Reduced Dorsolateral Prefrontal and Insular Cortex Activation to Food Images in Obesity: A Meta-Analysis of fMRI Studies, Plus one, 2013

25) U. Toepel et al. The brain tracks the energetic value in food images, NeuroImage, 2009

26) Metzger BE. International Association of Diabetes and Pregnancy Study Groups recommendations on the diagnosis and classification of hyperglycemia in pregnancy. Diabetes Care. 2010.

27) American Diabetes Association (ADA). 2. Classification and diagnosis of diabetes: Standards of medical care in diabetes-2021. Diabetes Care. 2021

28) Rasmussen, K. M., Catalano, P. M., & Yaktine, A. L. New guidelines for weight gain during pregnancy: what obstetrician/gynecologists should know. Current opinion in obstetrics & gynecology, 2009

29) Blechert, J., Lender, A., Polk, S., Busch, N. A., & Ohla, K. (2019). Food-Pics_Extended—An Image Database for Experimental Research on Eating and Appetite: Additional Images, Normative Ratings and an Updated Review. Frontiers in Psychology, 10 (307). doi:10.3389/fpsyg.2019.00307

30) Tylka, T. L., & Kroon Van Diest, A. M. (2013). The Intuitive Eating Scale-2: Item Refinement and Psychometric Evaluation With College Women and Men. Journal of Counseling Psychology, 60(1), 137–153. doi: 10.1037/a0030893

For peer review only - http://bmjopen.bmj.com/site/about/guidelines.xhtml

31) Camilleri, G. M., Méjean, C., Bellisle, F., Andreeva, V. A., Sautron, V., Hercberg, S., et al. (2015). Cross-cultural validity of the Intuitive Eating Scale-2. Psychometric evaluation in a sample of the general French population. Appetite, 84, 34–42. doi: 10.1016/j.appet.2014.09.009. Available from: http://dx.doi.org/10.1016/j.appet.2014.09.009

32) Karlsson J, Persson LO, Sjöström L, Sullivan M. Psychometric properties and factor structure of the Three-Factor Eating Questionnaire (TFEQ) in obese men and women. Results from the Swedish Obese Subjects (SOS) study. Int J Obes Relat Metab Disord. 2000 Dec;24(12):1715-25. doi: 10.1038/sj.ijo.0801442. PMID: 11126230.

33) Fleurbaix Laventie Ville Sante (FLVS) Study Group, The Three-Factor Eating Questionnaire-R18 Is Able to Distinguish among Different Eating Patterns in a General Population, The Journal of Nutrition, Volume 134, Issue 9, September 2004, Pages 2372–2380, https://doi.org/10.1093/jn/134.9.2372

34) Meule, A., Hermann, T., & Kübler, A. (2014). A short version of the food cravings questionnaire-trait: The FCQ-T-reduced. Frontiers in Psychology, 5(MAR). https://doi.org/10.3389/fpsyg.2014.00190

35) Brunault, P., el Archi, S., Ballon, N., Réveillère, C., & Barrault, S. (2018). Validation of the French version of the Food Cravings Questionaire-Trait-reduced: An easy-to-use and quick self-administered questionnaire to assess food craving. Annales Medico-Psychologiques, 176(8), 788–795. https://doi.org/10.1016/j.amp.2018.08.002

36) Cox JL, Holden JM, Sagovsky R. Detection of postnatal depression. Development of the 10-item Edinburgh postnatal depression scale. British Journal of Psychiatry 1987;150

37) Guedeney N, Fermanian J. Validation study of the French version of the Edinburgh postnatal depression scale (EPDS): new results about use and psychometric properties. European Psychiatry 1998;13:83–9.

38) Veale JF. Edinburgh Handedness Inventory - Short Form: a revised version based on confirmatory factor analysis. Laterality. 2014;19(2):164-77. doi: 10.1080/1357650X.2013.783045. Epub 2013 May 10. PMID: 23659650.

39) Crézé C, Candal L, Cros J, Knebel JF, Seyssel K, Stefanoni N, Schneiter P, Murray MM, Tappy L, Toepel U. The Impact of Caloric and Non-Caloric Sweeteners on Food Intake and Brain Responses to Food: A Randomized Crossover Controlled Trial in Healthy Humans. Nutrients. 2018 May 15;10(5):615. doi: 10.3390/nu10050615. PMID: 29762471; PMCID: PMC5986495.

40) Watson, D., Clark, L. A., & Tellegen, A. (1988). Development and Validation of Brief Measures of Positive and Negative Affect: The PANAS Scales. In Journal of Personality and Social Psychology (Vol. 54, Issue 6).

41) H. Caci, F. Baylé. L'Echelle d'Affectivité Positive et d'Affectivité Négative. Première traduction en français. Présenté à Congrès de l'Encéphale, Paris (2007).

42) Lesage, F. X., Berjot, S., & Deschamps, F. (2012). Psychometric properties of the french versions of the perceived stress scale. International Journal of Occupational Medicine and Environmental Health, 25(2), 178–184. https://doi.org/10.2478/S13382-012-0024-8

43) Meule, A. (2020). Twenty Years of the Food Cravings Questionnaires: a Comprehensive Review. In Current Addiction Reports (Vol. 7, Issue 1, pp. 30–43). Springer. https://doi.org/10.1007/s40429-020-00294-z

For peer review only - http://bmjopen.bmj.com/site/about/guidelines.xhtml

44) Blechert J, Goltsche JE, Herbert BM, Wilhelm FH. Eat your troubles away: electrocortical and experiential correlates of food image processing are related to emotional eating style and emotional state. Biol Psychol. 2014 Feb;96:94-101.

45) Cardi V, Leppanen J, Treasure J. The effects of negative and positive mood induction on eating behaviour: A meta-analysis of laboratory studies in the healthy population and eating and weight disorders. Neurosci Biobehav Rev. 2015 Oct;57:299-309.

46) Brunet, D., Murray, M.M., Michel, C.M., 2011. Spatiotemporal analysis of multichannel EEG: CARTOOL. Comput. Intell. Neurosci. doi:10.1155/2011/813870

47) Bohon C, Stice E, Spoor S. Female emotional eaters show abnormalities in consummatory and anticipatory food reward: a functional magnetic resonance imaging study. Int J Eat Disord. 2009 Apr;42(3):210-21. doi: 10.1002/eat.20615. PMID: 19040270; PMCID: PMC2739233.

48) Kostandyan M, Park HRP, Bundt C, González-García C, Wisniewski D, Krebs RM, Boehler CN. Are all behavioral reward benefits created equally? An EEG-fMRI study. Neuroimage. 2020 Jul 15;215:116829. doi: 10.1016/j.neuroimage.2020.116829. Epub 2020 Apr 10. PMID: 32283272.

For peer review only

For peer review only - http://bmjopen.bmj.com/site/about/guidelines.xhtml

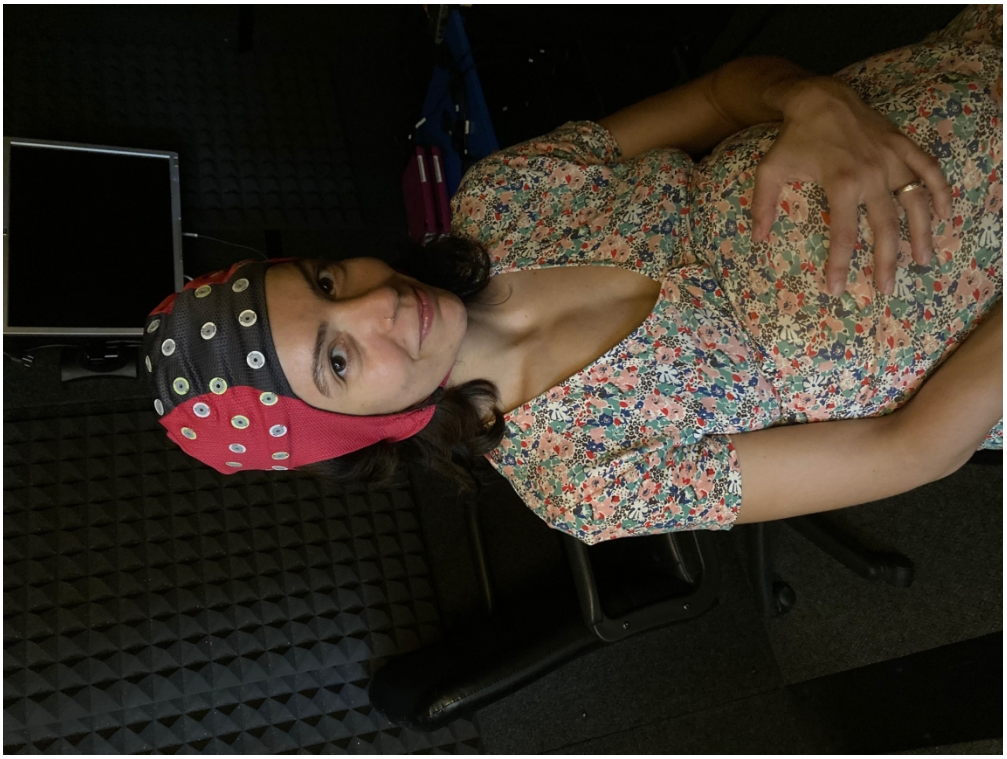

Figure 1. Electroencephalography

711x533mm (72 x 72 DPI)

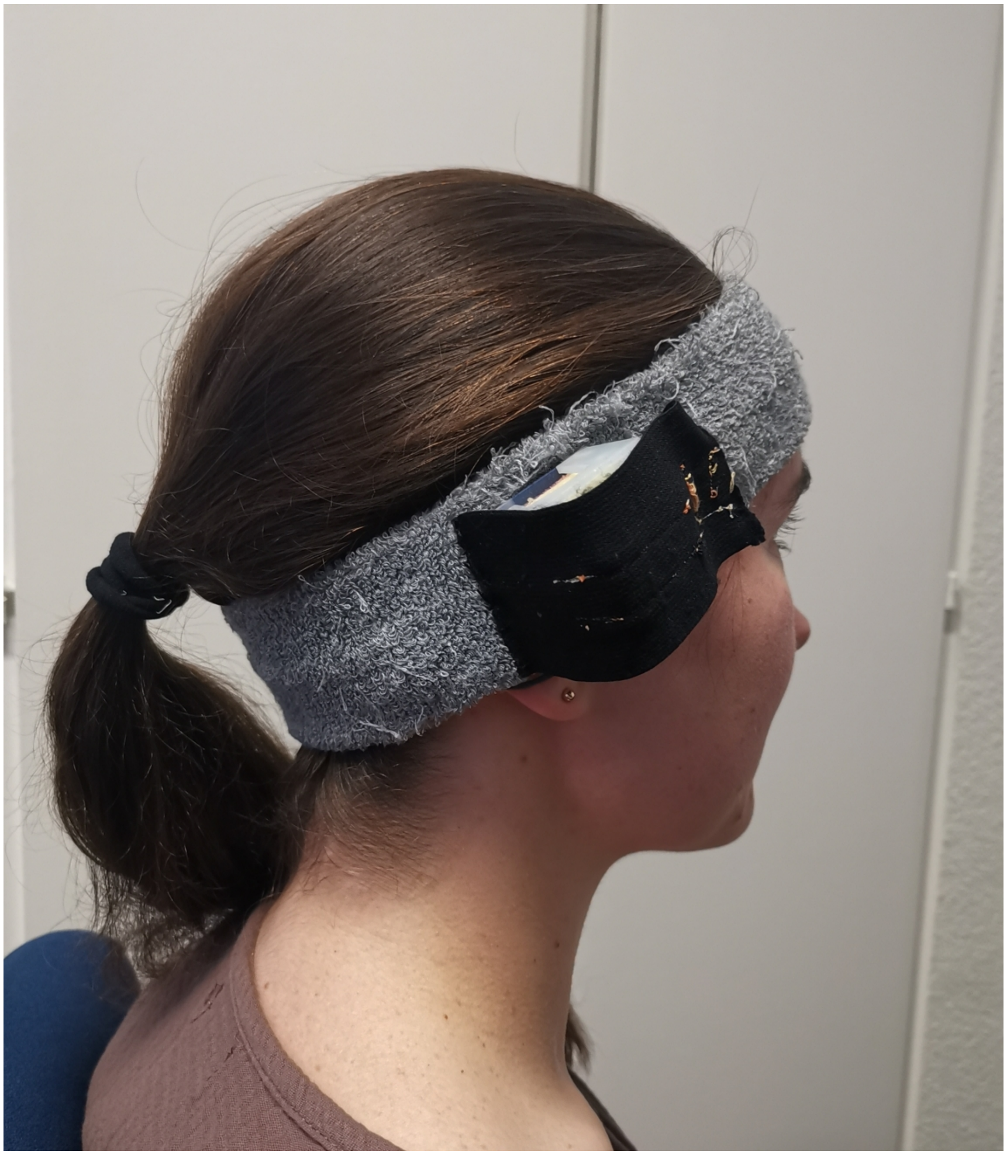

Figure 2. The Auracle

867x993mm (72 x 72 DPI)

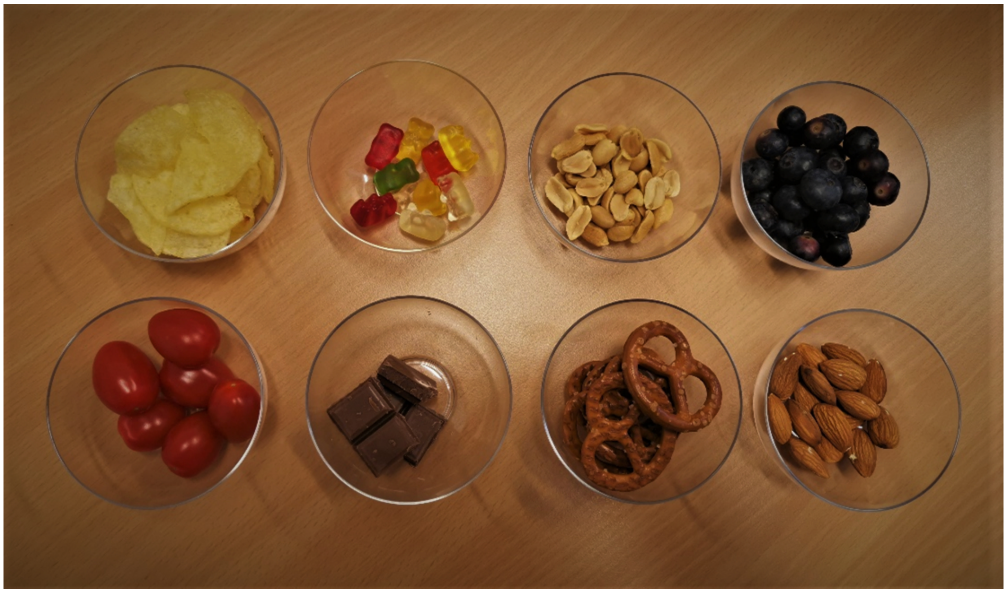

Figure 3. Snacks

324x190mm (87 x 87 DPI)

Brain responses to food viewing in women during pregnancy and postpartum and their relationship with metabolic health: Study protocol for the FOODY Brain Study, a prospective observational study.

Journal:	*BMJ Open*
Manuscript ID	bmjopen-2022-067013.R1
Article Type:	Protocol
Date Submitted by the Author:	31-Jan-2023
Complete List of Authors:	Lesniara-Stachon, Anna; Lausanne University Hospital, Woman-Mother-Child<br>Quansah, Dan Yedu; CHUV<br>Schenk, Sybille; CHUV<br>Retsa, Chrysa; Laboratory for Investigative Neurophysiology (the LINE), Radiology Department, Lausanne University Hospital and University of Lausanne, Lausanne, Switzerland<br>Halter, Ryan J; Thayer School of Engineering, Dartmouth College, Hanover, NH USA<br>Murray, Micah; Centre Hospitalier Universitaire Vaudois, Departments of Clinical Neurosciences and Department of Radiology<br>Lacroix, Alain; Institute of Higher Education and Research in Healthcare (IUFRS), University of Lausanne, Lausanne, Switzerland.<br>Horsch, Antje; University of Lausanne, Institute of Higher Education and Research in Healthcare; Lausanne University Hospital, Woman-Mother-Child<br>Toepel, Ulrike; Laboratory for Investigative Neurophysiology (the LINE), Radiology Department, Lausanne University Hospital and University of Lausanne, Lausanne, Switzerland<br>Puder, J; Universite de Lausanne, DFME
**Primary Subject Heading**:	Diabetes and endocrinology
Secondary Subject Heading:	Nutrition and metabolism, Mental health
Keywords:	Diabetes in pregnancy < DIABETES & ENDOCRINOLOGY, Neurobiology < NATURAL SCIENCE DISCIPLINES, NUTRITION & DIETETICS, Eating disorders < PSYCHIATRY

SCHOLARONE™ Manuscripts

For peer review only - http://bmjopen.bmj.com/site/about/guidelines.xhtml

TITLE: Brain responses to food viewing in women during pregnancy and postpartum and their relationship with metabolic health: Study protocol for the FOODY Brain Study, a prospective observational study

Anna Lesniara Stachon [1] (Anna.Lesniara-Stachon@chuv.ch), **Dan Yedu Quansah** [1] (Dan.Quansah@chuv.ch), **Sybille Schenk** [1,5] (Sybille.Schenk@chuv.ch), **Chrysa Retsa** [2,3,4] (chrysa.retsa@gmail.com), **Ryan J. Halter** [6,7] (Alain.Lacroix@chuv.ch), **Micah M. Murray** [2,3,4] (Micah.Murray@chuv.ch), **Alain Lacroix** [8,9] (Alain.Lacroix@chuv.ch), **Antje Horsch** [8,9] (Antje.Horsch@chuv.ch), **Ulrike Toepel** [2] (ulrike.toepel@unil.ch), **Jardena J. Puder** [1] (Jardena.Puder@chuv.ch)

[1] Obstetric Service, Department Woman-Mother-Child, Lausanne University Hospital, Lausanne, Switzerland

[2] Laboratory for Investigative Neurophysiology (the LINE), Radiology Department, Lausanne University Hospital and University of Lausanne, Lausanne, Switzerland

[3] The Sense Innovation and Research Center, Lausanne and Sion, Switzerland

[4] CIBM Center for Biomedical Imaging, Lausanne, Switzerland

[5] Service of Endocrinology, Diabetes and Metabolism, Lausanne University Hospital, Lausanne, Switzerland

[6] Thayer School of Engineering, Dartmouth College, Hanover, NH USA

[7] Department of Surgery, Geisel School of Medicine, Dartmouth College, Hanover, NH, USA

[8] Institute of Higher Education and Research in Healthcare (IUFRS), University of Lausanne, Lausanne, Switzerland

[9] Neonatology service, Department Woman-Mother-Child, Lausanne University Hospital, Lausanne, Switzerland

Correspondence to: Jardena J. Puder, Jardena.Puder@chuv.ch

For peer review only

ABSTRACT

Introduction: Food cravings are common in pregnancy and along with emotional eating and eating in the absence of hunger, they are associated with excessive weight gain and adverse effects on metabolic health including gestational diabetes mellitus (GDM). Women with GDM also show poorer mental health, which further can contribute to dysregulated eating behaviour. Food cravings can lead to greater activity in brain centers known to be involved in food 'wanting' and reward valuation as well as emotional eating. They are also related to gestational weight gain. Thus, there is a great need to link implicit brain responses to food with explicit measures of food intake behaviour, especially in the perinatal period. The aim of this study is to investigate the spatio-temporal brain dynamics to visual presentations of food in women during pregnancy and in the postpartum, and link these brain responses to the eating behaviour and metabolic health outcomes in women with and without GDM.

Methods and analysis: This prospective observational study will include 20 women with and 20 without GDM, that have valid data for the primary outcomes. Data will be assessed at 24-36 weeks gestational age and at 6 months postpartum. The primary outcomes are brain responses to food pictures of varying carbohydrate and fat content during pregnancy and in the postpartum using electroencephalography (EEG). Secondary outcomes including depressive symptoms, current mood,

and eating behaviours will be assessed with questionnaires, objective eating behaviours will be measured using Auracle and stress will be measured with heart rate and heart rate variability (Actiheart). Other secondary outcome measures include body composition and glycaemic control parameters.

Ethics and dissemination: The Human Research Ethics Committee of the Canton de Vaud approved the study protocol (2021-01976). Study results will be presented at public and scientific conferences and in peer-reviewed journals.

For peer review only

For peer review only - http://bmjopen.bmj.com/site/about/guidelines.xhtml

Strengths and limitations of this study:

1. This prospective observational study fills the knowledge gap regarding brain responses to food viewing during and after pregnancy.
2. This study includes subjective (questionnaires) and objective methodologies (EEG, Auracle device, Actiheart) to investigate brain responses to food viewing, eating behaviours and stress responses during and after pregnancy.
3. The study will link the outcomes of physiological and behavioral measures.
4. We will compare eating behaviours between healthy women and women with gestational diabetes, both cross-sectionally and prospectively.
5. Limitations include a small sample size (but comparable to other EEG studies with a similar design) and fact that all analyses (and the correlation of their outcomes) are exploratory, given the lack of data in pregnancy or the postpartum.

INTRODUCTION

Metabolic health and eating behaviours

The prevalence of obesity continues to increase worldwide (1). In the United States, 35.7% of women of reproductive age (20-39 years) are obese (2) and this prevalence exceeds 20% in high-income western countries in general (3).

The widespread and unrestricted access and intake of foods high in sugar and fat contribute to the obesity epidemic. Foods high in sugar (carbohydrates) and fats, such as chocolate, sweet desserts, and salty snacks are the most frequently craved foods (4). Sugar strongly stimulates the reward system, which can trigger compulsive eating (5). Eating behaviour refer to a variety of eating practices. This include cognitive restraint, disinhibition with high responsiveness to external food cues and susceptibility to hunger (6). Disinhibited eating behavior reflects a tendency to overeat. This can happen in the context of negative emotions (such as anxiety, depression, anger and loneliness) and/or in response to a high food palatability (7). Disinhibited eating behaviors can be associated with compulsive intakes of high energy-dense foods rich in fat and sugar which contribute to obesity and poorer long-term metabolic health (7). The ideal situation whereby eating behaviors are regulated by appetite and food consumption is challenged by disinhibited eating behaviors (8).

Another aspect of eating behaviour that relates to metabolic health is eating rate. Studies showed that faster eating rate is associated with increased food intake (9) and an increased risk of obesity (10). A meta-analyses of the cross-sectional and longitudinal association between eating speed and metabolic outcomes showed that faster eating rate is associated with higher risks for the metabolic syndrome, central obesity, elevated blood pressure and increased fasting plasma glucose compared to slower eating (11).

Brain regions in relationship to food cues

Although the underlying cause(s) of food cravings are not deeply understood, it has been shown in the general, non-pregnant population that cue-induced cravings in response to food pictures predict disordered eating and excess weight gain. In a meta-analytic review of 45 studies, exposure to food stimuli (pictures) and the experience of craving influence eating behaviour and further weight gain independently of baseline BMI, age and gender (12).

Some subjects are more susceptible to an obesogenic environment. This can be also mediated through different brain responses to food in key areas. The few existing clinical data show that

For peer review only - http://bmjopen.bmj.com/site/about/guidelines.xhtml

For peer review only

reward-related brain regions may be involved in excess weight gain (13). “The reward circuit is a complex neural network” (the cortico-ventral basal ganglia circuit), which consists, among other, of the orbitofrontal cortex , anterior cingulate cortex and the ventral striatum which is part of the basal ganglia (14). The ventral striatum receives information from the orbital prefrontal cortex, the insular cortex, the cingulate cortex and also the amygdala, which is also closely linked to sensory processing (14). These reward-related brain regions are stimulated when viewing the craved foods and through unconscious processes of learning/conditioning mechanisms, promote further greater ‘wanting’ and seeking of these foods (15). This could be related to the fact that higher intake of foods rich in fat and sugar can reduce reward thresholds in the limbic striatal pathways and the amygdala (15–17). This then promotes further food craving, further increases the neural reactivity to food cues in reward-related brain regions even more, and can thus promoting a higher intake of palatable foods whether in a hungry or satiated state (15,16,18). Investigating the brain's response to food with different fat and carbohydrate content may help to understand these mechanisms and their relationship to eating behaviour and metabolic health. So far, this has been mostly studied in healthy subjects.

Furthermore, physiological and metabolic parameters are also related to how the brain reacts to the sight of food. Response to viewing high- and low-energy foods differ between obese and healthy weight subjects (19). In fMRI study, overweight and obese participants demonstrated greater activation in reward-related brain regions, like the ventral striatum and anterior cingulate compared to normal weight subjects (19). Insulin resistance and type 2 diabetes mellitus (DM2) are associated with changes in brain responses to food stimuli (20). In patients with DM2, cross-sectional studies using functional magnetic resonance imaging (fMRI) revealed that compared to controls, responses to viewing food pictures are stronger in the ventral striatum and cortical regions, such as the insula and the orbitofrontal cortex, which are related to reward-related circuits, in comparison to controls (20). There is a need for prospective studies.

Stress, emotions and eating behavior

Emotions and stress can influence on food choices and eating behaviour (21,22). Studies showed that there is a link between negative mood, stress and the intake of high-fat, high-carbohydrate-hyperpalatabable food and higher overall food intake, which contribute to an increased risk for obesity (22,23). Food cravings and overeating in response to stress and negative mood are particularly relevant in people who engage in more frequent emotional eating (23–25). In a fMRI study, people who engage in more frequent emotional eating had stronger responses to high-calorie food in reward-associated areas (anterior cingulate cortex) when being in a negative mood in comparison to people who do not engage in more frequent emotional eating (26).

These results together demonstrate that there is a link between brain responses to food viewing and metabolic health, but also stress and emotions and that reward-related brain regions play a role in the regulation of food intake.

Pregnancy

Maintaining a healthy body weight is particularly important during pregnancy, as it impact on the health of the mother and her offspring (27). Maternal obesity and overnutrition during pregnancy affect the infant's metabolic programming and increase its risk of obesity, type 2 diabetes, and cardiovascular disease in the future (28).

Nevertheless, around 28% of women gain more weight than recommended (29) and excessive gestational weight gain (GWG) is associated with adverse perinatal outcomes such as preeclampsia, hypertension in pregnancy, gestational diabetes, preterm birth, cesarean section, and small or large

For peer review only - http://bmjopen.bmj.com/site/about/guidelines.xhtml

size for gestational age at birth (30). Higher GWG is also associated with greater postpartum weight retention (PPWR) and increasing the risk for future diabetes (31).

During pregnancy, there is a change in eating behaviour that can have long-term effects for the mother's metabolic health (32). Food cravings are frequent and concern around 40% of pregnant women (33). They typically begin at the end of the first trimester, peak in their frequency and intensity in the second and third trimester, and decrease after delivery (34). Higher food cravings during pregnancy have been associated with higher mean energy intake and GWG (33).

Emotions and stress also influence eating behaviour in pregnancy (35). Emotional cues such as negative affect can trigger the craving for high-fat foods in pregnant women which is associated with excessive GWG (8). Similarly, under stress, pregnant women are more likely to increase cravings for pleasurable food (36). There are no data regarding brain responses to food during pregnancy (using either MRI or EEG) or regarding the impact of emotions or stress on these brain responses.

Regarding metabolic health, insulin resistance increases in pregnancy and around 16% of pregnant women worldwide develop gestational diabetes mellitus (GDM) (37). GDM is defined as glucose intolerance with the first onset during pregnancy without fulfilling the criteria of overt diabetes (38). Women with GDM are at higher metabolic risk, have more perinatal complications such as preeclampsia or caesarean section (39) and also have a higher prevalence of mental health problems in pregnancy such as depression (40).

Postpartum period

There is a lack of data regarding eating behaviours in the postpartum and its relationship to PPWR (32). Existing studies showed that higher restrained and also higher intuitive eating are associated with postpartum weight loss (32). Knowledge related to cravings and emotional eating in the postpartum is limited. Negative emotions play a role in eating behaviour during this period, leading to increased food intake and contributing to PPWR (41). Regarding the group of women of higher metabolic risk, those with GDM have more adverse maternal health outcomes in the postpartum such as higher PPWR, a 7-10 fold higher risk of diabetes (42) and an increased risk for cardiovascular disease (43,44). GDM is also associated with higher risk of postpartum depression (45) which is also associated with a less healthy diet (46).

Electroencephalography

EEG and visual evoked potentials (VEPs; patterns of electrical activity in the brain in response to a visual stimulus) to food viewing offer the advantage of disentangling the temporal as well as spatial dynamics of perception, while fMRI only unravels spatial aspects, i.e., brain regions. Some VEP studies have shown differential responses to pictures of high and low-energy foods (47). Reward- and (inhibitory) control-related processes are partially recruiting the same neural sources, yet with varying temporal dynamics (48). Existing studies on food cravings during and after pregnancy only assessed different kinds of food cravings and their frequency with questionnaires and did not involve brain responses to food cues. Results from these studies have been inconsistent. We are not aware of any human studies investigating objective correlates or mechanistic pathways of cravings in pregnancy. In order to better understand food cravings and metabolic health outcomes in pregnancy and postpartum, investigating the neural activities of the brain pathways in response to cravings induced by food viewing during and after pregnancy might lead to novel clinical treatment options.

Overall, the relationship between weight, GWG, PPWR and adverse perinatal and metabolic health outcomes in the perinatal period and the difficulty to reduce weight or weight gain, highlight the

For peer review only - http://bmjopen.bmj.com/site/about/guidelines.xhtml

need for novel approaches to understand eating behaviour and metabolic health during pregnancy and the postpartum, especially in metabolic high-risk women with GDM.

As there are no data regarding brain responses to food in pregnancy and in the postpartum, we designed an observational prospective study whose design is exploratory. Nevertheless, based on the above mentioned data, we hypothesized that brain responses, especially to food high in fat and carbohydrate content would differ between pregnancy and the postpartum and between healthy women and women at higher metabolic risk such as GDM. We also hypothesized that there would be a relationship between brain responses to viewing food with different fat and carbohydrate content, eating behavior and metabolic health and that negative emotions and higher stress levels might influence these brain responses as well as eating behavior in the perinatal period.

Aims of the present study

The overall aim of this observational study is to investigate brain responses to viewing pictures of foods of different fat and carbohydrate contents in women during pregnancy and in the postpartum and their relationship with eating behaviour and metabolic health. We will investigate this in healthy women and in a metabolic high-risk group, i.e., women with GDM to include a wide spectrum of metabolic health.

The main objectives are:

Objective 1: To investigate differences in brain responses to food pictures of varying fat and carbohydrate content (HIGH-FAT/LOW-CARB; HIGH-FAT/HIGH-CARB; LOW-FAT/LOW-CARB; LOW-FAT/HIGH-CARB) during and after pregnancy, in women with and without GDM.

Objective 2: To investigate the cross-sectional and longitudinal relationships between brain responses to food viewing with reported food cravings, eating behaviour, and metabolic health outcomes such as weight, body fat and glucose control during pregnancy and in the postpartum.

Objective 3: To investigate differences in brain responses to food pictures in relationship to different emotional states and stress levels.

METHODS

Study design

This is a prospective observational study of women with and without GDM. There will be two visits (at 24-36 weeks gestational age and 6 months postpartum). Both visits will take place at Lausanne University Hospital.

Study population, inclusion and exclusion criteria, and recruitment

We will include 40 women with valid data for the primary outcomes (20 women with GDM and 20 healthy controls) at both time points. In the initial tests, the EEG data of 13 women with GDM and 12 healthy controls were not valid (i.e too much noise, low quality of signal), which has been corrected since. We will therefore recruit a total 33 women with GDM and 32 healthy controls, in their 24-36 weeks of pregnancy, during their antenatal appointments visits at a Swiss University Hospital. GDM women (clinical group) will be recruited at the GDM clinic whereas healthy pregnant women without GDM (control group) will be recruited at the hospital's ambulatory clinic.

For women with GDM, inclusion criteria are: gestational age 24- 36 weeks, age ≥ 18 years, GDM diagnosed at 24-32 gestational weeks according to IADPSG and ADA criteria (49,50). For healthy controls, inclusion criteria include women with normal weight (pre-pregnancy BMI < 25 kg/m2), with

For peer review only - http://bmjopen.bmj.com/site/about/guidelines.xhtml

no prior or current GDM, no diets and weight changes above 5 kg in the last 5 years (except for pregnancy), no excessive pregnancy weight changes (less than twice of the Institute of Medicine recommendations (51). Women in both groups should be able to fluently speak and write either French or English.

Exclusion criteria include: pre-existing diabetes, eating disorders (Anorexia Nervosa, Bulimia Nervosa, and Binge-Eating Disorder), vegetarian/vegan diet, uncontrollable nausea and vomiting, current insulin treatment, participation in an intervention study, current medication to treat psychiatric disorders, active suicidal thoughts.

Primary outcome

The primary outcomes are EEG viewing responses, specifically to food images depending on fat and carbohydrate content (HIGH-FAT/LOW-CARB; HIGH-FAT/HIGH-CARB; LOW-FAT/LOW-CARB; LOW-FAT/HIGH-CARB). These responses will be examined in terms of amplitude and latency of the brain responses (VEPs) as well as in terms of response topography. An estimation of the underlying intracranial sources of the VEPs will also be performed. The full analysis pipeline is referred to as "Electrical Neuroimaging" as has been developed and validated by members of our team and their collaborators (for example, Michel and Murray, 2012; Murray et al., 2008; Brunet et al., 2011). VEPs will be compared during (visit 1) and after (visit 2) pregnancy, as well as between women with and without GDM.

Secondary outcomes

Secondary outcomes are weight, HbA1c, glucose measures at visits 1 and 2 (the latter only in women with GDM) and body composition (fat/lean mass). We will investigate eating rate, objectively using a novel eating recognition device (Auracle) and subjectively (reported perceived eating rate). Using validated self-report questionnaires we will assess eating behaviours, including cravings (Food Craving Questionnaire-Trait-reduced), intuitive eating (IE) (The French Intuitive Eating Scale-2), external, emotional eating, and cognitive restriction (The Three-Factor Eating Questionnaire). Depression scores will be assessed by Edinburgh Postnatal Depression Scale and current mood by PANAS questionnaire Positive and Negative Affective Schedule. Other measures will include self-reported stress level assessed with a questionnaire (The Perceived Stress Scale) and objectively measures of stress using Actiheart.

Data collection and visits

The study started in March 2022 and the end of the study is scheduled for June 2024.

The first visit will take place during pregnancy, at 24-36 weeks gestational age (visit 1) and the second visit at 6 months postpartum (visit 2).

After signing the informed consent, and before visit 1 and visit 2, participants will complete validated questionnaires online (see below). They will be contacted by phone to complete missing data regarding their medical history as well as their lifestyle behaviours.

Before each visit, participants will be asked to eat a balanced meal around 12.00 (based on the Swiss Society for Nutrition recommendations; the patients are given examples and at the end will be checked what they eat) to minimize the bias of different satiety level, will come to the hospital at 13h30 and testing will be completed at 16h (Table 1).

For peer review only - http://bmjopen.bmj.com/site/about/guidelines.xhtml

Flowchart of the study procedure:

			Visit 1	Visit 2
	Study schedule			
Time point			3rd trimester of pregnancy	6 month postpartum
Duration			Tele-visit: 10-30 min for medical history and 20-40 min for online questionnaires<br><br>Visit: max 2h 30 min including breaks*	Tele-visit: 10-30 min for medical history and 20-40 min for online questionnaires<br><br>Visit: max 2h 30 min including breaks*
Procedures and time				
**Tele-visit before or after each of both of the physical visits (at home)**				
Medical history if lacking in the chart and questions regarding lifestyle, by phone		10-30 min		
Questionnaires online: Food Craving Questionnaire-Trait-reduced, Edinburgh Postnatal Depression Scale, Edinburgh Handedness Inventory, TFEQ - R18, Intuitive Eating Scale – 2		20-40 min	X	X
**During the visit (in the EEG lab up to the break)**				
Set-up EEG, questionnaires:<br><br>Perceived Stress Scale (PSS-10)<br>Food Craving Questionnaire-State<br>PANAS questionnaire: Positive and Negative Affective Schedule,<br>current hunger level: Likert scale) + heart rate (Actiheart)	13h30-14h15	30 - 45 min	X	X

For peer review only - http://bmjopen.bmj.com/site/about/guidelines.xhtml

Electroencephalography	14h15-15h10	55 min	X	X
Break	15h10-15h20	10 min	X	X
Bioimpedance & weight	15h20-15h30	10 min	X	X
Positive and Negative Affective Schedule current hunger level: Likert scale<br><br>Break and Auracle* + questionnaire: Eating of the provided snacks & 3 questions regarding eating duration	15h30-15h45	15 min	X	X
Height, blood pressure	15h45-15h50	5 min	X	X
Capillary measure: HbA1c Double check lunch composition	15h50-15h55	5 min	X	X
Set-up glucose sensor: Freestyle Libre 1 (only for GDM) x 14 days	15h55-16h00	5 min	X	X

Table 1: Study schedule.

**During the Auracle session, patients will eat different snacks that we provide.*

Patient and Public Involvement

The development of the research question and outcome measures was also based on remarks from patients that were followed in clinical settings and we informally asked their advice while designing the study. Some results will be provided to participants at the end of their study as feedback by sending them to their healthcare providers. Interested patients will also receive an overall return of the results.

MEASURES

Behavioural task/Electroencephalography

We will employ a behavioural task involving food pictures of variable fat and carbohydrate content and will record high-density VEPs (brain responses to visual stimuli via electroencephalography (EEG)) (figure 1). Using the Salzburg food picture database (52), we are going to show 4 blocks of 240 randomized images (200 pictures of food and 40 non-food objects together; 50 pictures of food and 10 non-food objects per block) to the participants. The food images are divided into 4 categories: HIGH-FAT/LOW-CARB; HIGH-FAT/HIGH-CARB; LOW-FAT/LOW-CARB; LOW-FAT/HIGH-CARB.

HIGH-FAT was defined by a fat content of > 5 g/100g (21.6 +/- 13.77 g/100g) and LOW-FAT of < 5 g/100g (mean 1.1 +/- 1.29 g/100g). Similarly, HIGH-CARB was defined as >15g/100g carb (48.57 +/-

For peer review only - http://bmjopen.bmj.com/site/about/guidelines.xhtml

21.13 g/100g) and LOW-CARB as < 15 g/100g (4.59 +/- 4.59 g/100g). We aimed for the contents to be as far as possible away from the cut-off to provide the best distinction.

Images will be identical in size, controlled for low-level visual features (like luminance), and will be presented on the computer monitor during EEG recording sessions. Immediately after the presentation of each image, women have to decide if the presented picture is a food or non-food object by pressing a button 1 or 2 on a response box. Accuracy and reaction time of behavioural responses will be collected. At the beginning of the experimental session, the participant will be presented with the instructions and will have to perform some trials (7 pictures of food and 3 of non-food) to familiarize herself with the task. Continuous 128-channel EEG will be recorded on the head surface. VEPs will be calculated separately for each image category (HIGH-FAT/LOW-CARB; HIGH-FAT/HIGH-CARB; LOW-FAT/LOW-CARB; LOW-FAT/HIGH-CARB). The preparation for the EEG recording will take approximately 30-45 min, whilst EEG sessions will last maximum 55 minutes. When the EEG experiment is completed, the patient will move to the preparation room for the validation task. Twenty pictures of food (5 pictures from each food category) will be presented and the patient will be asked to rate how much she wants this food. During visit 2, 4 blocks with randomised images of food and non-food objects will be presented in a different order than during visit 1 to minimise potential sources of bias.

Heart rate and heart rate variability

During EEG and the Auracle testing, we will monitor the participant's heart rate and heart rate variability to assess the level of arousal of the autonomic system. We will perform baseline and recovery measurements (2 min each) before and after EEG and the Auracle testing. For this purpose, we will use Actiheart, a compact chest-worn monitoring tool that apart from heart rate, measures Inter-Beat-Interval (IBI). Specific time points before and after the test will be identified by a stopwatch. To analyze the data, we will use the commercial Actiheart Software and Kubios.

Body composition

Body composition will be measured by bioimpedance (BIA 101, Akern, Italy) at both visits. Using four electrodes (two on the right hand and two on the right foot, at a distance of 3–4 cm from each other) and low-voltage electric flows, we will measure the resistance (Rz) and reactance (Xc) of tissue to assess the fat and lean mass. We will also use BIA InBody s10 to measure body composition by bioimpedance at both visits. The body composition analysis of InBody S10 is derived from the 4-compartment model, which include Total Body Water, Protein, Mineral and Body Fat (53).

Eating behaviour ("Auracle") and choice of snacks

The Auracle (see Figure 2) will be used as an objective measure of eating behaviour during snack session (see Figure 3). Auracle is an eating-recognition system that measures vibrations generated from movement, chewing and teeth grinding. When worn, the contact microphone is positioned on the mastoid and when a person eats, vibrations from movement, chewing and grinding of teeth sensed by the device are transmitted and stored on an embedded memory card. This device automatically identifies the eating rate and rapidity as well as the duration of food intake and other related dynamics with an accuracy exceeding 92% (54). Auracle provides a holistic, detailed and accurate characterization of objective correlates of eating behavior. All of the sensors in Auracle (ADXL362 accelerometer and CM-01B microphone) are commercial, off-the-shelf devices. The device includes an MSP430 microcontroller (Texas Instruments, Dallas TX), a push button and buzzer for user interaction. It is comfortable to wear because it is embedded in an elastic headband or a flexible plastic frame and possesses no risk to the mother or fetus. The Auracle device has been validated and has been used in in healthy non-pregnant subjects (54,55).

For peer review only - http://bmjopen.bmj.com/site/about/guidelines.xhtml

Eight different crunchy snacks of different carbohydrate and fat content (2 of each corresponding to one of the four categories described in the EEG part) will be offered (Figure 3). As eating behaviour will be measured with the "Auracle" device, crunchy-textured snacks provide a stronger, more easily detected vibrational signal. Before the snack session, the patient will be asked the second time about their current level of hunger (1- not at all to 5- very hungry) and fill out the "PANAS" questionnaire. The weight of the different snacks will be measured before and after the patient has finished eating to determine the number of kcal and the quantity of fat, protein and carbohydrates eaten. Furthermore, the time interval between the first and last bite will be also measured using the "Auracle" device and a stopwatch. We will also ask the woman questions regarding her subjective perceived eating rate (perceived eating duration, perceived eating speed and current eating speed compared to usual eating speed).

Collection of blood samples

Capillary blood will be collected to measure HbA1c level. HbA1c will be analyzed by Afinion®.

Glucose sensors

Women with GDM will use Freestyle Libre 1 glucose sensors (Abbott®) for flash glucose measurement for 14 days. Sensors will be applied on the back of the arm, with a sterile fiber being inserted under the skin into the interstitial fluid. Patients use a dedicated application LibreLink and will have to scan the glucose sensor at least every 8h to transfer data. Data will be linked in a coded form to the online platform Libreview (https://www.libreview.com/). During pregnancy, results will be discussed with their health care provider during the visits. For the postpartum visit, patients will receive feedback by phone.

Questionnaires related to eating behavior and mental health

Women with and without GDM will fill out the same questionnaires during both visits (visit 1 & 2). Most women will fill out the questionnaires in French and a few in English, as around 80% of patients that participate in our studies are French-speaking. The following questionnaires will be filled out online before the study visits:

The French Intuitive Eating Scale-2 (IES-2) (56): Validated self-report questionnaire that assesses individuals' tendency to follow their physical, hunger and satiety cues in determining when, what and how much to eat. The original version (English) consists of 4 subscales, Eating for physical rather than emotional reasons (EPR, 8 items), Reliance on hunger and satiety cues (RHSC, 6 items), Unconditional permission to eat (UPE, 6 items) and the Body-Food Choice Congruence (BFC-C, 3 items) subscales. The validated French version however consists of 18-items divided into three subscales, the EPR (8-items), RHSC (6-items) and the UPE (4-items) subscales (57), and has also demonstrated good psychometric properties. In this study, we will assess intuitive eating with only the EPR and the RHSC subscales (14-items) of the French-adapted version of IES-2 and the English version. Scores of IES-2 range between 1 and 5. A higher score of the EPR subscale reflects eating as an answer to hunger and a lower score means eating to cope with emotional distress, whereas a higher score on the RHSC subscale signifies trust in internal cues, and a lower score reflects less ability to regulate food intake.

The Three-Factor Eating Questionnaire-Revised 18 (TFEQ-R18): 18-item self-report questionnaire that measures three aspects of eating behaviours including cognitive restraint (6 items), uncontrolled eating (9 items), and emotional eating (3 items). Items are scored on a Likert scale ranging from 1 'Almost always' to 4 'Never'. A short, revised version of the Stunkard and Messick TFEQ was developed by Karlsson et al. (58). It has been also tested in the general population in France (59).

For peer review only - http://bmjopen.bmj.com/site/about/guidelines.xhtml

Food Craving Questionnaire-Trait-reduced (FCQ-T-r) (60): 15 item self-report questionnaire for the assessment of the frequency and intensity of food craving. It consists of 5 subscales: lack of control over eating (5 items), thoughts or preoccupation with food (5 items), intentions and plans to consume food (2 items), emotions before or during food craving (2 items), and cues that may trigger food craving (1 item). Items are scored on a Likert scale from 1 'Never/not applicable' to 6 'Always'. Higher scores reflect food-cue elicited craving. The French version was validated and has shown good psychometric proprieties (61).

Edinburgh Postnatal Depression Scale (EPDS) (62): 10-item self-report questionnaire to examine postnatal depression symptoms over the previous week, which are scored on a 4-point Likert scale. Scores range from 0 to 30. Higher scores indicate a higher risk of postnatal depression. The French version of the EPDS demonstrated good psychometric properties (63).

Edinburgh Handedness Inventory short form (64): This short (4 items) and simple questionnaire measures the preference to use one hand more than the other. The French version has been previously used in research (65).

During the study visits, participants will complete the following questionnaires:

The Positive and Negative Affective Schedule questionnaire (PANAS) (66) : Self-report questionnaire that assesses mood or emotion during the last month. It consists of 20 items, 10 items measuring positive affect (e.g., enthusiastic, proud) and 10 items measuring negative affect (e.g., scared, ashamed), which are scored on a Likert scale from 1 'Not at All' to 5 'Extremely'. Individual scores on the PANAS range from 10 to 50, with higher scores representing higher levels of positive affect. The validated French version has shown good psychometrics (67).

The Perceived Stress Scale (PSS-10): 10 item questionnaire widely used to assess stress levels during the last month. Responses are rated on a Likert scale from 0 'Never' to 5 'Very often'. The results range from 0 to 40, with higher scores indicating higher perceived stress. The French version of PSS-10 has shown good psychometric properties (68) .

The Food Craving Questionnaire-State (FCQ–S) (69): 15 item questionnaire measures the intensity of momentary food cravings. Items are scored on a 5-point scale ranging from 'strongly disagree' to 'strongly agree'. Higher scores indicate more intense current food cravings. The French version was translated and used in previous research by the research team of our collaborator Ulrike Toepel (65)

The current hunger level scale: This is one item developed for this study assessing the current perceived hunger level using a Likert scale ranging from 1 'just a little/not at all' to 5 'very much'.

The food wanting questionnaire: 20-item questionnaire was developed for this study and will be completed after the EEG (see above). Women will be shown 20 pictures of food and asked 'How much do you want this food?'. They will then have to indicate their response on a Likert scale from 0 'not at all' to 10 'very much'.

DATA MANAGEMENT AND STATISTICAL ANALYSES

Statistical analysis plan

The sample size is based on previous EEG studies of similar design (23,25), but all analyses are exploratory, as to our knowledge so far no such study has been conducted during pregnancy or the postpartum period. Statistical analyses will be performed with STATA version 15.1 (2017) and MATLAB version 9.5 (R2018b). Processing of the EEG data will be performed using Cartool software and statistical software (70). Demographic and other descriptive variables will be represented as

For peer review only - http://bmjopen.bmj.com/site/about/guidelines.xhtml

means and standard deviations or percentages where appropriate. ANOVA, t-tests or Chi-square tests will be used to determine group differences between responses to food and non-food pictures, different food categories, brain responses between pregnancy and postpartum states, between women with and without GDM and between normal weight and overweight/obese women. Regression analyses will be used to model the relationships between predictor variables and outcomes (such as between brain responses to food viewing with intuitive eating and metabolic health such as weight, body fat and glucose control). In the second step, we will investigate the impact of covariates, where appropriate, such as maternal age, gestational age, and pre-pregnancy BMI on outcomes and adjust for them if necessary.

Twenty women with valid data for the primary endpoint in each group (n=40) will be investigated during pregnancy and at 6 months postpartum. In order to achieve this, we had to include more patients.

Ethics and dissemination

The local ethical committee approved the study protocol (study number 2021-01976). The study poses little to no risk to participants. Signed informed consent will be obtained from all participating women. The results of the study will be disseminated through national and international conferences, in peer-reviewed journals and presented at public conferences and other scientific meetings.

Significance and outlook

The prevalence of obesity continues to increase worldwide. This study seeks to understand brain reactions to the viewing of food images and their changes during pregnancy and in the postpartum period. Food cravings and disordered eating behaviors include implicit processes in the brain. Studying these processes could help unravel mechanistic pathways associated with weight gain. Our study may provide novel evidence of scientific and clinical importance to understand eating behaviour, potentially opening perspectives for future interventions that alter reactions to energy-dense food in general or at specific food nutrients and/or brain regions to manage excessive GWG, weight retention, and to reduce adverse metabolic outcomes in the postpartum period. It is a potential opportunity to reduce craving, incentive sensitization and thus the overstimulation of reward circuits by food cues in pregnant and postpartum states where a dynamic change in craving and weight gain is observed. The study aims to provide data for a better understanding and long-lasting interventions that can influence the health of the mother and the child.

Author affiliations

[1] Obstetric Service, Department Woman-Mother-Child, Lausanne University Hospital, Lausanne, Switzerland

[2] Laboratory for Investigative Neurophysiology (the LINE), Radiology Department, Lausanne University Hospital and University of Lausanne, Lausanne, Switzerland

[3] The Sense Innovation and Research Center, Lausanne and Sion, Switzerland

[4] CIBM Center for Biomedical Imaging, Lausanne, Switzerland

[5] Service of Endocrinology, Diabetes and Metabolism, Lausanne University Hospital, Lausanne, Switzerland

[6] Thayer School of Engineering, Dartmouth College, Hanover, NH USA

[7] Department of Surgery, Geisel School of Medicine, Dartmouth College, Hanover, NH, USA

For peer review only - http://bmjopen.bmj.com/site/about/guidelines.xhtml

[8] Institute of Higher Education and Research in Healthcare (IUFRS), University of Lausanne, Lausanne, Switzerland.

[9] Neonatology service, Department Woman-Mother-Child, Lausanne University Hospital, Lausanne, Switzerland.

Author contributions JP designed the study with input from all co-authors, particularly DQ. CHR, UT and MMM contributed to the creation and design of the EEG section, along with SS, DQ, ALS and JP. SS was responsible for the nutritional part regarding the lunch and snacks. AH contributed to the psychology part and AL, along with DQ, to the statistical and data analysis part. RJH was responsible for the design of "Auracle", adapting it together with DQ. ALS wrote the first draft and all authors critically revised the final version of the manuscript.

Funding This work was supported by a donation from the Dreyfus foundation and an unrestricted educational grant from NovoNordisk. JJ Puder's research is funded by the Leenaards price in translational medical research (2019, Grant Number 4522.8). Ryan Halter's research program is supported by the US National Science Foundation under award numbers CNS-1565269, CNS-1835983, CNS-1565268, and CNS-1835974.

Competing interest None declared.

Patient consent for publication Not required.

Ethics approval Commission d'Ethique du Canton de Vaud, Switzerland, study number: 2021-01976

Open access This is an open access article distributed in accordance with the Creative Commons Attribution Non Commercial (CC BY-NC 4.0) license, which permits others to distribute, remix, adapt, build upon this work non-commercially, and license their derivative works on different terms, provided the original work is properly cited, appropriate credit is given, any changes made indicated, and the use is non-commercial. See: http://creativecommons.org/licenses/by-nc/4.0/.

Figure legends

Figure 1: 128-channel electroencephalography, Figure 2: The Auracle: eating recognition system, Figure 3: Eight snacks of different carbohydrate and fat content

For peer review only - http://bmjopen.bmj.com/site/about/guidelines.xhtml

REFERENCES

1. Abarca-Gómez L, Abdeen ZA, Hamid ZA, Abu-Rmeileh NM, Acosta-Cazares B, Acuin C, et al. Worldwide trends in body-mass index, underweight, overweight, and obesity from 1975 to 2016: a pooled analysis of 2416 population-based measurement studies in 128·9 million children, adolescents, and adults. The Lancet. 2017 Dec;390(10113):2627–42.

2. Hales CM, Fryar CD, Carroll MD, Freedman DS, Ogden CL. Trends in Obesity and Severe Obesity Prevalence in US Youth and Adults by Sex and Age, 2007-2008 to 2015-2016. JAMA. 2018 Apr 24;319(16):1723.

3. Iurilli ML, Zhou B, Bennett JE, Carrillo-Larco RM, Sophiea MK, Rodriguez-Martinez A, et al. Heterogeneous contributions of change in population distribution of body mass index to change in obesity and underweight. Elife. 2021 Mar 9;10.

4. Gilhooly CH, Das SK, Golden JK, McCrory MA, Dallal GE, Saltzman E, et al. Food cravings and energy regulation: the characteristics of craved foods and their relationship with eating behaviors and weight change during 6 months of dietary energy restriction. Int J Obes. 2007 Dec 26;31(12):1849–58.

5. Wang GJ. Impact of sugar on the body brain and behavior. Frontiers in Bioscience. 2018;23(12):4704.

6. Stunkard AJ, Messick S. The three-factor eating questionnaire to measure dietary restraint, disinhibition and hunger. J Psychosom Res. 1985 Jan;29(1):71–83.

7. Bryant EJ, King NA, Blundell JE. Disinhibition: its effects on appetite and weight regulation. Obesity Reviews. 2007 Dec 26;9(5):409–19.

8. Blau LE, Orloff NC, Flammer A, Slatch C, Hormes JM. Food craving frequency mediates the relationship between emotional eating and excess weight gain in pregnancy. Eat Behav. 2018 Dec;31:120–4.

9. Ohkuma T, Hirakawa Y, Nakamura U, Kiyohara Y, Kitazono T, Ninomiya T. Association between eating rate and obesity: a systematic review and meta-analysis. Int J Obes. 2015 Nov 25;39(11):1589–96.

10. Wuren, Endoh K, Kuriki K. Eating rate as risk for body mass index and waist circumference obesity with appropriate confounding factors: a cross-sectional analysis of the Shizuoka-Sakuragaoka J-MICC Study. Asia Pac J Clin Nutr. 2019;28(1):79–91.

11. Yuan S qian, Liu Y ming, Liang W, Li F fei, Zeng Y, Liu Y yue, et al. Association Between Eating Speed and Metabolic Syndrome: A Systematic Review and Meta-Analysis. Front Nutr. 2021 Oct 20;8.

12. Boswell RG, Kober H. Food cue reactivity and craving predict eating and weight gain: a meta-analytic review. Obesity Reviews. 2016 Feb;17(2):159–77.

13. Frankort A, Roefs A, Siep N, Roebroeck A, Havermans R, Jansen A. The Craving Stops Before You Feel It: Neural Correlates of Chocolate Craving During Cue Exposure with Response Prevention. Cerebral Cortex. 2014 Jun;24(6):1589–600.

14. Haber SN. Neuroanatomy of Reward: A View from the Ventral Striatum. 2011.

For peer review only - http://bmjopen.bmj.com/site/about/guidelines.xhtml

15. Sinha R. Role of addiction and stress neurobiology on food intake and obesity. Biol Psychol. 2018 Jan;131:5–13.

16. Cottone P, Sabino V, Roberto M, Bajo M, Pockros L, Frihauf JB, et al. CRF system recruitment mediates dark side of compulsive eating. Proceedings of the National Academy of Sciences. 2009 Nov 24;106(47):20016–20.

17. Ghitza UE, Gray SM, Epstein DH, Rice KC, Shaham Y. The Anxiogenic Drug Yohimbine Reinstates Palatable Food Seeking in a Rat Relapse Model: a Role of CRF1 Receptors. Neuropsychopharmacology. 2006 Oct 7;31(10):2188–96.

18. McCrickerd K, Forde CG. Sensory influences on food intake control: moving beyond palatability. Obesity Reviews. 2016 Jan 11;17(1):18–29.

19. Jensen CD, Kirwan CB. Functional brain response to food images in successful adolescent weight losers compared with normal-weight and overweight controls. Obesity. 2015 Mar;23(3):630–6.

20. Chechlacz M, Rotshtein P, Klamer S, Porubská K, Higgs S, Booth D, et al. Diabetes dietary management alters responses to food pictures in brain regions associated with motivation and emotion: a functional magnetic resonance imaging study. Diabetologia. 2009 Mar 13;52(3):524–33.

21. Macht M, Simons G. Emotions and eating in everyday life. Appetite. 2000 Aug;35(1):65–71.

22. Yau YHC, Potenza MN. Stress and Eating Behaviors. 2014.

23. Cardi V, Leppanen J, Treasure J. The effects of negative and positive mood induction on eating behaviour: A meta-analysis of laboratory studies in the healthy population and eating and weight disorders. Neurosci Biobehav Rev. 2015 Oct;57:299–309.

24. Yau YHC, Potenza MN. Stress and eating behaviors. Minerva Endocrinol. 2013 Sep;38(3):255–67.

25. Blechert J, Goltsche JE, Herbert BM, Wilhelm FH. Eat your troubles away: Electrocortical and experiential correlates of food image processing are related to emotional eating style and emotional state. Biol Psychol. 2014 Feb;96:94–101.

26. Bohon C, Stice E, Spoor S. Female emotional eaters show abnormalities in consummatory and anticipatory food reward: A functional magnetic resonance imaging study. International Journal of Eating Disorders. 2009 Apr;42(3):210–21.

27. Kominiarek MA, Peaceman AM. Gestational weight gain. Am J Obstet Gynecol. 2017 Dec;217(6):642–51.

28. Dyer J, Rosenfeld C. Metabolic Imprinting by Prenatal, Perinatal, and Postnatal Overnutrition: A Review. Semin Reprod Med. 2011 May 18;29(03):266–76.

29. Martínez-Hortelano JA, Cavero-Redondo I, Álvarez-Bueno C, Garrido-Miguel M, Soriano-Cano A, Martínez-Vizcaíno V. Monitoring gestational weight gain and prepregnancy BMI using the 2009 IOM guidelines in the global population: a systematic review and meta-analysis. BMC Pregnancy Childbirth. 2020 Dec 27;20(1):649.

For peer review only - http://bmjopen.bmj.com/site/about/guidelines.xhtml

30. Voerman E, Santos S, Inskip H, Amiano P, Barros H, Charles MA, et al. Association of Gestational Weight Gain With Adverse Maternal and Infant Outcomes. JAMA. 2019 May 7;321(17):1702.

31. Catalano PM, Shankar K. Obesity and pregnancy: mechanisms of short term and long term adverse consequences for mother and child. BMJ. 2017 Feb 8;j1.

32. Bijlholt M, van Uytsel H, Ameye L, Devlieger R, Bogaerts A. Eating behaviors in relation to gestational weight gain and postpartum weight retention: A systematic review. Obesity Reviews. 2020 Oct;21(10).

33. Hill AJ, McCance DR. Anthropometric and nutritional associations of food cravings in pregnancy. Pregnancy Hypertension: An International Journal of Women's Cardiovascular Health. 2014 Jul;4(3):235.

34. Belzer LM, Smulian JC, Lu SE, Tepper BJ. Food cravings and intake of sweet foods in healthy pregnancy and mild gestational diabetes mellitus. A prospective study. Appetite. 2010 Dec;55(3):609–15.

35. Chang MW, Nitzke S, Guilford E, Adair CH, Hazard DL. Motivators and Barriers to Healthful Eating and Physical Activity among Low-Income Overweight and Obese Mothers. J Am Diet Assoc. 2008 Jun;108(6):1023–8.

36. Mooney J, Lipsky LM, Liu A, Nansel TR. Does stress attenuate motivation for healthful eating in pregnancy and postpartum? Appetite. 2021 Aug;163:105207.

37. Plows J, Stanley J, Baker P, Reynolds C, Vickers M. The Pathophysiology of Gestational Diabetes Mellitus. Int J Mol Sci. 2018 Oct 26;19(11):3342.

38. 2. Classification and Diagnosis of Diabetes: *Standards of Medical Care in Diabetes—2020*. Diabetes Care. 2020 Jan 1;43(Supplement_1):S14–31.

39. Tan PC, Ling LP, Omar SZ. The 50-g glucose challenge test and pregnancy outcome in a multiethnic Asian population at high risk for gestational diabetes. International Journal of Gynecology & Obstetrics. 2009 Apr;105(1):50–5.

40. OuYang H, Chen B, Abdulrahman AM, Li L, Wu N. Associations between Gestational Diabetes and Anxiety or Depression: A Systematic Review. J Diabetes Res. 2021 Jul 27;2021:1–10.

41. O'Loghlen E, Galligan R. Disordered eating in the postpartum period: Role of psychological distress, body dissatisfaction, dysfunctional maternal beliefs and self-compassion. J Health Psychol. 2022 Apr 16;27(5):1084–98.

42. Bellamy L, Casas JP, Hingorani AD, Williams D. Type 2 diabetes mellitus after gestational diabetes: a systematic review and meta-analysis. The Lancet. 2009 May;373(9677):1773–9.

43. Kramer CK, Campbell S, Retnakaran R. Gestational diabetes and the risk of cardiovascular disease in women: a systematic review and meta-analysis. Diabetologia. 2019 Jun 7;62(6):905–14.

44. Gunderson EP, Sun B, Catov JM, Carnethon M, Lewis CE, Allen NB, et al. Gestational Diabetes History and Glucose Tolerance After Pregnancy Associated With Coronary Artery Calcium in Women During Midlife. Circulation. 2021 Mar 9;143(10):974–87.

For peer review only - http://bmjopen.bmj.com/site/about/guidelines.xhtml

45. Wilson CA, Newham J, Rankin J, Ismail K, Simonoff E, Reynolds RM, et al. Is there an increased risk of perinatal mental disorder in women with gestational diabetes? A systematic review and meta-analysis. Diabetic Medicine. 2020 Apr 29;37(4):602–22.

46. Gilbert L, Gross J, Lanzi S, Quansah DY, Puder J, Horsch A. How diet, physical activity and psychosocial well-being interact in women with gestational diabetes mellitus: an integrative review. BMC Pregnancy Childbirth. 2019 Dec 7;19(1):60.

47. Toepel U, Knebel JF, Hudry J, le Coutre J, Murray MM. The brain tracks the energetic value in food images. Neuroimage. 2009 Feb;44(3):967–74.

48. Kostandyan M, Park HRP, Bundt C, González-García C, Wisniewski D, Krebs RM, et al. Are all behavioral reward benefits created equally? An EEG-fMRI study. Neuroimage. 2020 Jul;215:116829.

49. 2. Classification and Diagnosis of Diabetes: *Standards of Medical Care in Diabetes—2021*. Diabetes Care. 2021 Jan 1;44(Supplement_1):S15–33.

50. International Association of Diabetes and Pregnancy Study Groups Recommendations on the Diagnosis and Classification of Hyperglycemia in Pregnancy. Diabetes Care. 2010 Mar 1;33(3):676–82.

51. Rasmussen KM, Catalano PM, Yaktine AL. New guidelines for weight gain during pregnancy: what obstetrician/gynecologists should know. Curr Opin Obstet Gynecol. 2009 Dec;21(6):521–6.

52. Blechert J, Lender A, Polk S, Busch NA, Ohla K. Food-Pics_Extended—An Image Database for Experimental Research on Eating and Appetite: Additional Images, Normative Ratings and an Updated Review. Front Psychol. 2019 Mar 7;10.

53. https://inbodyusa.com/products/inbodys10/.

54. Bi S, Wang T, Davenport E, Peterson R, Halter R, Sorber J, et al. Toward a Wearable Sensor for Eating Detection. In: Proceedings of the 2017 Workshop on Wearable Systems and Applications. New York, NY, USA: ACM; 2017. p. 17–22.

55. Bi S. Detection of health-related behaviors using head-mouted devices. Dartmouth College. 2021.

56. Tylka TL, Kroon Van Diest AM. The Intuitive Eating Scale–2: Item refinement and psychometric evaluation with college women and men. J Couns Psychol. 2013;60(1):137–53.

57. Camilleri GM, Méjean C, Bellisle F, Andreeva VA, Sautron V, Hercberg S, et al. Cross-cultural validity of the Intuitive Eating Scale-2. Psychometric evaluation in a sample of the general French population. Appetite. 2015 Jan;84:34–42.

58. Karlsson J, Persson LO, Sjöström L, Sullivan M. Psychometric properties and factor structure of the Three-Factor Eating Questionnaire (TFEQ) in obese men and women. Results from the Swedish Obese Subjects (SOS) study. Int J Obes. 2000 Dec 4;24(12):1715–25.

59. Blandine de Lauzon, Romon M, Deschamps V, Lafay L, Borys JM, Karlsson J, et al. The Three-Factor Eating Questionnaire-R18 Is Able to Distinguish among Different Eating Patterns in a General Population. J Nutr. 2004 Sep 1;134(9):2372–80.

For peer review only - http://bmjopen.bmj.com/site/about/guidelines.xhtml

60. Meule A, Hermann T, KÃ¼bler A. A short version of the Food Cravings Questionnaireâ€"Trait: the FCQ-T-reduced. Front Psychol. 2014 Mar 4;5.

61. Brunault P, el Archi S, Ballon N, Réveillère C, Barrault S. Validation de la version française du Food Cravings Questionnaire-Trait-reduced : un auto-questionnaire simple et de passation rapide pour mesurer le craving alimentaire. Annales Médico-psychologiques, revue psychiatrique. 2018 Oct;176(8):788–95.

62. Cox JL, Holden JM, Sagovsky R. Detection of Postnatal Depression. British Journal of Psychiatry. 1987 Jun 2;150(6):782–6.

63. Guedeney N, Fermanian J. Validation study of the French version of the Edinburgh Postnatal Depression Scale (EPDS): new results about use and psychometric properties. European Psychiatry. 1998 Apr 16;13(2):83–9.

64. Veale JF. Edinburgh Handedness Inventory – Short Form: A revised version based on confirmatory factor analysis. Laterality: Asymmetries of Body, Brain and Cognition. 2014 Mar 4;19(2):164–77.

65. Crézé C, Candal L, Cros J, Knebel JF, Seyssel K, Stefanoni N, et al. The Impact of Caloric and Non-Caloric Sweeteners on Food Intake and Brain Responses to Food: A Randomized Crossover Controlled Trial in Healthy Humans. Nutrients. 2018 May 15;10(5):615.

66. Watson D, Clark LA, Tellegen A. Development and validation of brief measures of positive and negative affect: The PANAS scales. J Pers Soc Psychol. 1988;54(6):1063–70.

67. H. Caci FB. L'Echelle d'Affectivité Positive et d'Affectivité Négative. Première traduction en français. Présenté à Congrès de l'Encéphale. In Paris; 2007.

68. Lesage FX, Berjot S, Deschamps F. Psychometric properties of the French versions of the Perceived Stress Scale. Int J Occup Med Environ Health. 2012 Jan 1;25(2).

69. Meule A. Twenty Years of the Food Cravings Questionnaires: a Comprehensive Review. Curr Addict Rep. 2020 Mar 12;7(1):30–43.

70. Brunet D, Murray MM, Michel CM. Spatiotemporal Analysis of Multichannel EEG: CARTOOL. Comput Intell Neurosci. 2011;2011:1–15.

For peer review only

For peer review only - http://bmjopen.bmj.com/site/about/guidelines.xhtml

For peer review only

For peer review only - http://bmjopen.bmj.com/site/about/guidelines.xhtml

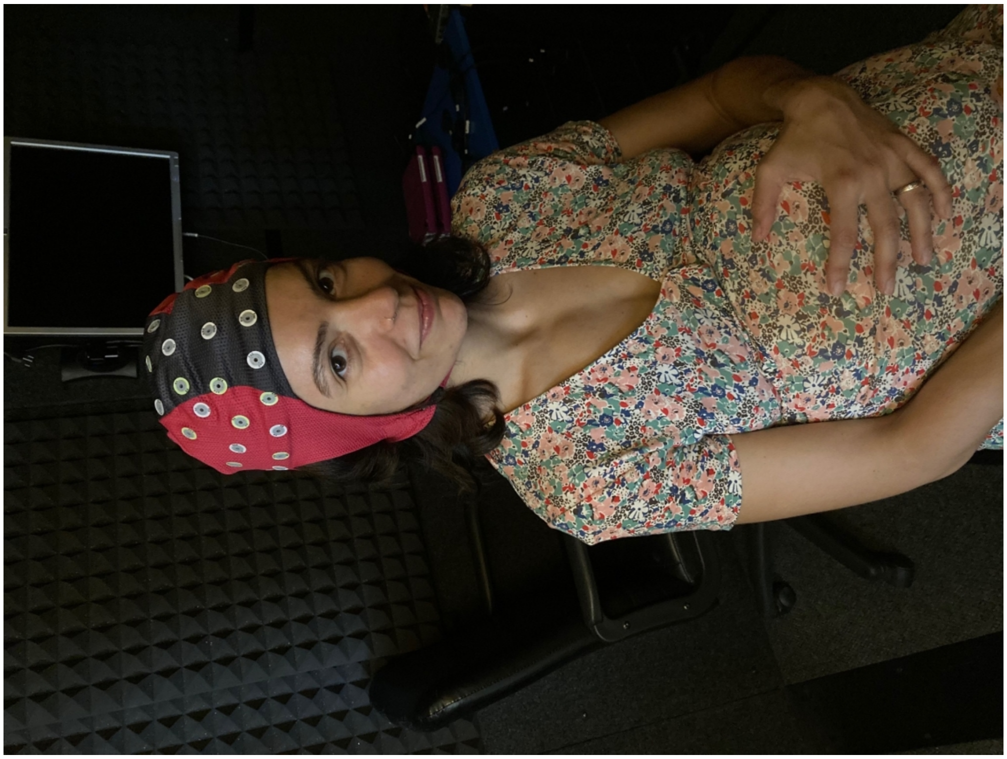

128-channel electroencephalography

711x533mm (72 x 72 DPI)

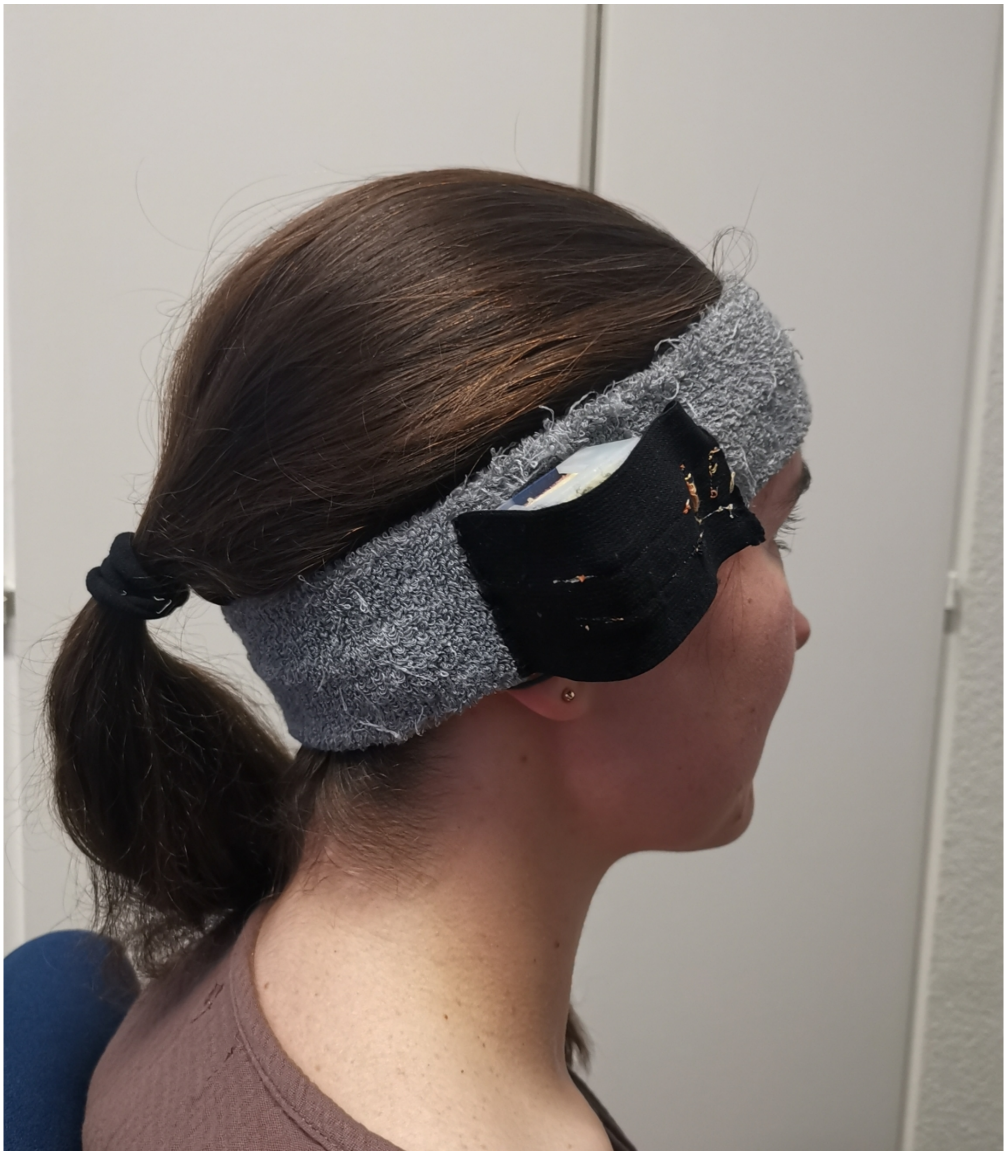

The Auracle: eating recognition system

867x993mm (72 x 72 DPI)

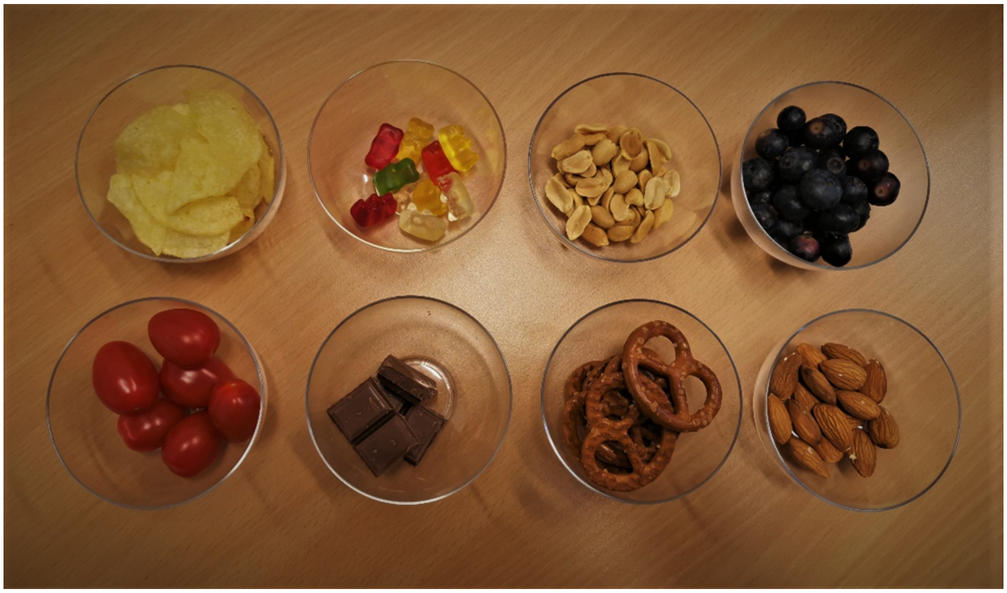

Eight snacks of different carbohydrate and fat content

324x190mm (87 x 87 DPI)

STROBE Statement—checklist of items that should be included in reports of observational studies

	Item No.	Recommendation	Page No.	Relevant text from manuscript
**Title and abstract**	1	(*a*) Indicate the study's design with a commonly used term in the title or the abstract	1	"Study protocol for the FOODY Brain Study, a prospective observational study"
		(*b*) Provide in the abstract an informative and balanced summary of what was done and what was found		n/a
**Introduction**				
Background/rationale	2	Explain the scientific background and rationale for the investigation being reported	3-5	The scientific background and rationale are explained in the subsections: "Metabolic health and eating behaviours", "Brain regions in relationship to food cues", "Stress, emotions and eating behavior", "Pregnancy", "Postpartum period" and "Electroencephalography".
Objectives	3	State specific objectives, including any prespecified hypotheses	6	"We hypothesized that brain responses, especially to food high in fat and carbohydrate content would differ between pregnancy and the postpartum and between healthy women and women at higher metabolic risk such as GDM. We also hypothesized that there would be a relationship between brain responses to viewing food with different fat and

For peer review only - http://bmjopen.bmj.com/site/about/guidelines.xhtml

				carbohydratecontent, eating behavior and metabolic health and that negative emotions and higher stress levels might influence these brain responses as well as eating behavior in the perinatal period." Aims are included in the subsection: "Aims of the present study".
**Methods**				
Study design	4	Present key elements of study design early in the paper	6	At the beginning of the methods part, there is subsection: "Study design"<br>"This is a prospective observational study of women with and without GDM. There will be two visits (at 24-32 weeks gestational age and 6 months postpartum). Both visits will take place at Lausanne University Hospital."
Setting	5	Describe the setting, locations, and relevant dates, including periods of recruitment, exposure, follow-up, and data collection	7	"Data collection and visits":<br>The study started in March 2022 and the end of the study is scheduled for June 2024.<br>The first visit will take place during pregnancy, at 24-36 weeks gestational age (visit 1) and the second visit at 6 months postpartum (visit 2).

For peer review only - http://bmjopen.bmj.com/site/about/guidelines.xhtml

For peer review only

				After signing the informed consent, and before visit 1 and visit 2, participants will complete validated questionnaires online (see below). They will be contacted by phone to complete missing data regarding their medical history as well as their lifestyle behaviours.<br>Before each visit, participants will be asked to eat a balanced meal around 12.00 (based on the Swiss Society for Nutrition recommendations), will come to the hospital at 13h30 and testing will be completed at 16h (Table 1)."
Participants	6	(*a*) *Cohort study*—Give the eligibility criteria, and the sources and methods of selection of participants. Describe methods of follow-up<br>*Case-control study*—Give the eligibility criteria, and the sources and methods of case ascertainment and control selection. Give the rationale for the choice of cases and controls<br>*Cross-sectional study*—Give the eligibility criteria, and the sources and methods of selection of participants	6-7	All required information of the cohort are in section "Study population, inclusion and exclusion criteria, and recruitment" and "Data collection and visits" of our manuscript.
		(*b*) *Cohort study*—For matched studies, give matching criteria and number of exposed and unexposed<br>*Case-control study*—For matched studies, give matching criteria and the number of controls per case	n/a	Cohort study, but subjects are not matched. Numbers of recruited participants are mentioned in the "Study population, inclusion and exclusion criteria, and recruitment" section.

For peer review only - http://bmjopen.bmj.com/site/about/guidelines.xhtml

Variables	7	Clearly define all outcomes, exposures, predictors, potential confounders, and effect modifiers. Give diagnostic criteria, if applicable	7, 13	All required information about outcomes are in section: “Primary outcome” and “Secondary outcomes” of our manuscript. Information about potential confounders can be found in the “Statistical analysis plan”: “We will investigate the impact of covariates, such as maternal age, gestational age, and pre-pregnancy BMI on outcomes and adjust for them if necessary.”
Data sources/ measurement	8*	For each variable of interest, give sources of data and details of methods of assessment (measurement). Describe comparability of assessment methods if there is more than one group	9-12	We described methods of measurements in details in section “Methods” of our manuscript We did it separately for each variable of interest in the following subsections: “Behavioural task/Electroencephalography”, “Heart rate and heart rate variability”, “Body composition”, “Collection of blood samples”, ” Glucose sensors”, “Questionnaires related to eating behaviour and mental health”.
Bias	9	Describe any efforts to address potential sources of bias	7, 10	We addressed potential sources of bias in the “Data collection and visits” section: “Before

For peer review only - http://bmjopen.bmj.com/site/about/guidelines.xhtml

				each visit, participants will be asked to eat a balanced meal around 12.00 (based on the Swiss Society for Nutrition recommendations) to minimize the bias of different satiety level" and in the "Behavioural task/Electroencephalography" section: "During visit 2, 4 blocks with randomised images of food and non-food objects will be presented in a different order than during visit 1 to minimise potential sources of bias"
Study size	10	Explain how the study size was arrived at	12	In the subsection "Statistical analysis plan", we added information: "The sample size is based on previous EEG studies of similar design (22,24), but all analyses are exploratory, as to our knowledge so far no such study has been conducted during pregnancy or the postpartum period."

Continued on next page

For peer review only - http://bmjopen.bmj.com/site/about/guidelines.xhtml

For peer review only

Quantitative variables	11	Explain how quantitative variables were handled in the analyses. If applicable, describe which groupings were chosen and why	12-13	We described statistical methods in the subsection "Statistical analysis plan".
Statistical methods	12	(*a*) Describe all statistical methods, including those used to control for confounding	12-13	We described statistical methods in the subsection "Statistical analysis plan".
		(*b*) Describe any methods used to examine subgroups and interactions	12-13	We described statistical methods in the subsection "Statistical analysis plan".
		(*c*) Explain how missing data were addressed	n/a	
		(*d*) *Cohort study*—If applicable, explain how loss to follow-up was addressed<br>*Case-control study*—If applicable, explain how matching of cases and controls was addressed<br>*Cross-sectional study*—If applicable, describe analytical methods taking account of sampling strategy	n/a	
		(*e*) Describe any sensitivity analyses	n/a	
**Results**				
Participants	13*	(a) Report numbers of individuals at each stage of study—eg numbers potentially eligible, examined for eligibility, confirmed eligible, included in the study, completing follow-up, and analysed	n/a	
		(b) Give reasons for non-participation at each stage	n/a	
		(c) Consider use of a flow diagram	n/a	
Descriptive data	14*	(a) Give characteristics of study participants (eg demographic, clinical, social) and information on exposures and potential confounders	n/a	
		(b) Indicate number of participants with missing data for each variable of interest	n/a	
		(c) *Cohort study*—Summarise follow-up time (eg, average and total amount)	n/a	
Outcome data	15*	*Cohort study*—Report numbers of outcome events or summary measures over time	n/a	
		Case-control study—Report numbers in each exposure category, or summary measures of exposure	n/a	
		Cross-sectional study—Report numbers of outcome events or summary measures	n/a	
Main results	16	(*a*) Give unadjusted estimates and, if applicable, confounder-adjusted estimates and their precision (eg, 95% confidence interval). Make clear which confounders were adjusted for and why they were included	n/a	
		(*b*) Report category boundaries when continuous variables were categorized	n/a	

For peer review only - http://bmjopen.bmj.com/site/about/guidelines.xhtml

	(*c*) If relevant, consider translating estimates of relative risk into absolute risk for a meaningful time period	n/a

Continued on next page

For peer review only

Other analyses	17	Report other analyses done—eg analyses of subgroups and interactions, and sensitivity analyses	n/a	
**Discussion**				
Key results	18	Summarise key results with reference to study objectives	n/a	
Limitations	19	Discuss limitations of the study, taking into account sources of potential bias or imprecision. Discuss both direction and magnitude of any potential bias	n/a	
Interpretation	20	Give a cautious overall interpretation of results considering objectives, limitations, multiplicity of analyses, results from similar studies, and other relevant evidence	n/a	
Generalisability	21	Discuss the generalisability (external validity) of the study results	n/a	
**Other information**				
Funding	22	Give the source of funding and the role of the funders for the present study and, if applicable, for the original study on which the present article is based	14	"Funding This work was supported by a donation from the Dreyfus foundation and an unrestricted educational grant from NovoNordisk. JJ Puder's research is funded by the Leenaards price in translational medical research (2019, Grant Number 4522.8). Ryan Halter's research program is supported by the US National Science Foundation under award numbers CNS-1565269, CNS-1835983, CNS-1565268, and CNS-1835974."

*Give information separately for cases and controls in case-control studies and, if applicable, for exposed and unexposed groups in cohort and cross-sectional studies.

Note: An Explanation and Elaboration article discusses each checklist item and gives methodological background and published examples of transparent reporting. The STROBE checklist is best used in conjunction with this article (freely available on the Web sites of PLoS Medicine at http://www.plosmedicine.org/, Annals of Internal Medicine at http://www.annals.org/, and Epidemiology at http://www.epidem.com/). Information on the STROBE Initiative is available at www.strobe-statement.org.

For peer review only - http://bmjopen.bmj.com/site/about/guidelines.xhtml
